# Genome-wide analysis of trehalose-6-phosphate phosphatases (TPP) gene family in wheat indicates their roles in plant development and stress response

**DOI:** 10.1186/s12870-022-03504-0

**Published:** 2022-03-16

**Authors:** Linying Du, Shumin Li, Li Ding, Xinxiu Cheng, Zhensheng Kang, Hude Mao

**Affiliations:** 1 State Key Laboratory of Crop Stress Biology for Arid Areas, College of Life Science, Northwest A&F University, Yangling, 712100 Shaanxi China; 2 State Key Laboratory of Crop Stress Biology for Arid Areas, College of Plant Protection, Northwest A&F University, Yangling, 712100 Shaanxi China; 3 College of Plant Science, Tarim University, Alar, Xinjiang, 843300 China

**Keywords:** *TaTPPs*, Phylogenetic relationship, Expression profiles, Plant development, Stress response, *TaTPP11*

## Abstract

**Background:**

Trehalose-6-phosphate phosphatases genes (*TPPs*) are involved in the development and stress response of plants by regulating the biosynthesis of trehalose, though little is currently known about *TPPs* in common wheat (*Triticum aestivum* L.).

**Results:**

In this study, we performed a genome-wide identification of the *TPP* gene family in common wheat, and identified a total of 31 *TaTPP* genes. These were subdivided into six subfamilies based on the phylogenetic relationships and the conservation of protein in six monocot and eudicot plants. The majority of *TPP* genes were represented by 2–3 wheat homoalleles (named *TaTPPX_ZA*, *TaTPPX_ZB*, or *TaTPPX_ZD*), where Z is the location on the wheat chromosome of the gene number (X). We also analyzed the chromosomal location, exon-intron structure, orthologous genes, and protein motifs of the *TaTPPs*. The RNA-seq data was used to perform an expression analysis, which found 26 *TaTPP* genes to be differentially expressed based on spatial and temporal characteristics, indicating they have varied functions in the growth and development of wheat. Additionally, we assessed how the promoter regulatory elements were organized and used qRT-PCR in the leaves to observe how they were expressed following ABA, salt, low tempreture, and drought stress treatments. All of these genes exhibited differential expression against one or more stress treatments. Furthermore, ectopic expression of *TaTPP11* in *Arabidopsis* exhibited a phenotype that delayed plant development but did not affect seed morphology.

**Conclusions:**

*TaTPPs* could serve important roles in the development and stress response in wheat. These results provide a basis for subsequent research into the function of *TaTPP*s.

**Supplementary Information:**

The online version contains supplementary material available at 10.1186/s12870-022-03504-0.

## Background

Improving crop yields under both positive and negative field conditions is needed to increase worldwide food security. However, increasing the resilience and potential of crop yields at the same time is difficult, since the factors that responsible for stress tolerance and productivity are typically at odds with each other [1]. Trehalose, a non-reducing disaccharide, is found in algae, invertebrates, bacteria, plants, fungi, and invertebrates [2]. The fact that trehalose is present in such a variety of life forms, coupled with the various biosynthetic pathways, indicates that trehalose metabolism serves an important evolutionary role by guarding the structure of cells and bioactive materials (including nucleic acids, membranes, and proteins) under environmental stressors (including freezing, oxidative, low-temperature, high-temperature, high-saline, and drought conditions) [3–5].

Plants typically only accumulate a small amount of trehalose and its intermediates, so it unlikely to play an osmoprotective role [4]. Instead, the trehalose metabolic pathway and its related intermediates detect and regulate energetic status of cells [2, 6]. For example, when it is exogenously applied, trehalose changes the enzymes involved in the accumulation of storage carbohydrates in photosynthetic tissues as well as its gene expression (including the induction of *AGPase* genes found in *Arabidopsis* [7]), and increases the drought tolerance and biomass yield [8–10]. In addition, previous study have revealed that the trehalose pathway is involved in the early stages of seed germination in *Medicago truncatula* during seed imbibition with water or stress agents (polyethylene glycol and sodium chloride), the trehalose was significantly reduced during seed absorption was measured by HPLC (high performance liquid chromatography), indicating that trehalose may be an energy source rather than an osmotic protector [11]. Moreover, The *ramosa3* mutant of *Zea mays* have significantly reduced trehalose and results excessive branching [12, 13]. Trehalose-6-phosphate (T6P) is an intermediate of the trehalose metabolic pathway and serves an important roles during the signaling of plant sugars, assists in the regulation of the use and allotment of sucrose, and regulates the growth and development of crops [2, 6, 14–16]. For example, inducing an increase in T6P decreases the degradation of starch in *Arabidopsis*, while T6P alterations regulate flowering patterns and the photoperiod [17]. Recent studies have demonstrated that the relationship between SnRK1 (SNF1-related/AMPK protein kinases) and T6P pathways can significantly alter how carbon is used and allotted in plants. T6P and SnRK1 pathways play opposite roles in metabolism control and T6P inhibits SnRK1 in several plant tissues. Specifically, raising the levels of T6P can induce flux via the biosynthetic pathways responsible for yield and growth, while lowering the levels of T6P can mobilize carbon stores and induce the transport of carbon related to stress response [1, 2, 15, 18]. Similar studies also demonstrated that T6P appear to have a functional role in the regulation of SnRK1 kinase activity by inhibiting SnRK1 physiological amounts (1–100 μM) as well as in a tissue- and developmental stage-specific manner [19, 20]. Hence, T6P can be targeted at particular types of tissues and cells during specific developmental stages to increase the resilience and potential of crop yields.

Plants synthesize trehalose by way of a conserved, two-step metabolic pathway. The first step entails the catalyzation of glucose from UDP-glucose to glucose 6-phosphate (G6P) via trehalose-6-phosphate synthase (TPS), resulting in trehalose-6-phosphate (T6P). The T6P is then dephosphorylated into trehalose via trehalose-6-phosphate phosphatase (TPP) [21]. The *TPP* and *TPS* genes have been found in species from all major plants, suggesting that the metabolism of trehalose is likely found throughout the plant kingdom [7, 22–24]. So far, there are eleven *TPS* genes were encoded by the rice and *Arabidopsis* genomes, while 10 and 13 *TPP* genes were encoded by the rice and *Arabidopsis* genomes, respectively [25, 26]. TPP proteins in all plants are comprised of a particular TPP domain that has conserved phosphatase domains, while all encode functional TPP enzymes in *Arabidopsis*. Furthermore, TPP share similar activities but differ in their patterns of differential expression, suggesting they could have a function related to specific tissues, stages, or processes [27].

A few *TPP* genes have recently been associated with abiotic stress responses. *AtTPPD* is a plastidial isoform regulated by redox reactions and associated with oxidative stress and salt resistance in *Arabidopsis* [24], while *AtTPPF* and *AtTPPI* are two isoforms associate with drought response [28, 29]. *OsTPP1* and *OsTPP2* were induced by cold stress in rice [30, 31], while the *OsTPP7* gene helps resist anaerobiosis during the germination stage in rice, and this trait has been lost from many kinds of commercial varieties [32]. The *MADS6* promoter is active during the flowering of reproductive tissue and contributes to the expression of the *OsTPP1* gene. This allows for significant improvements in both grain set and yield during the flowering stage under various drought conditions [14]. In addition, a number of TPP proteins serve important roles during plant development. For example, losing maize *RAMOSA3* and *ZmTPP4* reduces the determination of the meristem and increases inflorescence branching [12, 33].

Bread wheat (*Triticum aestivum* L*.*; 2n = 6x = 42; AABBDD) is widely grown and eaten around the globe [34]. The *TPP* genes play important roles in the development and stress response in plants. Therefore, we conducted a genome-wide analysis of the identification and expression of *TPP* genes in wheat. Firstly, a phylogenetic tree was produced to assess the evolutionary relationships of TPP with wheat and other plants. Then, we analyzed the conserved motifs, gene structures of TaTPPs. Besides, expression patterns of *TaTPPs* were also analyzed in the stems, leaves, flag leaves, roots, spikes and grains across various developmental stages. Further, the *cis*-regulatory elements of the promoter sequences of *TaTPPs* and the expression level of *TaTPPs* in ABA, salt, low tempreture, and drought stress treatments were analyzed and detected in wheat. Finally, we found that *TaTPP11* overexpression in *Arabidopsis* exhibited a developmentally delayed phenotype compared with wild-type plants. In summary, this study provides a basis for subsequent research on the function of *TaTPP* genes.

## Results

### Identification of the *TPP* gene family in wheat

We obtained the wheat genome data from the Chinese Spring IWGSC RefSeq v1.1 reference genome assembly (https://wheat-urgi.versailles.inra.fr/). Firstly, a UNIX pipeline was used to convert the wheat genome to a local BLAST database. Then, 23 TPP protein sequences from *Arabidopsis* and rice were employed to execute a BLAST search (BLASTP) with the local blast database, using a cut-off *E*-value <1e^− 10^. After filtering redundant sequences, we analyzed the remaining protein sequences and identify the TPP domain by the Simple Modular Architecture Research Tool (SMART; http://smart.embl-heidelberg.de/smart/ set_mode.cgi?NORMAL = 1). Finally, 31 TPP domain containing proteins were identified in the most recent wheat genome (Additional file 1: Table S1). However, when compare to the 33 wheat TPP members identified by Paul et al [1], we found TraesCS3D02G488100 and TraesCS6B02G156900 are not have the TPP domain, so we removed them from the *TaTPP* gene family. Of these 31 wheat TPP members, we assigned 11 clusters to various A, B, or D sub-genomes, which we considered to be homologous copies of a single *TPP* gene. Wheat *TPP* genes were named as *TaTPPX_ZA*, *TaTPPX_ZB*, or *TaTPPX_ZD*, and Z denote the location on the wheat chromosome where the gene number (X) is located. The detailed information of *TaTPP* genes in wheat was listed in Table 1. As shown in Table 1, the identified *TaTPP* genes in wheat encode proteins ranging from 249 (TaTPP5-2A) to 584 (TaTPP7-3D) amino acids (aa) in length with an average of 386 aa. Furthermore, the computed molecular weights of these TaTPP proteins ranged from 28.66 (TaTPP5-2A) to 96.02 (TaTPP7-3D) kDa. The theoretical pI of the TaTPP proteins ranged from 5.53 (TaTPP1-1B and TaTPP1-1D) to 9.26 (TaTPP10-6A).

**Table 1:** Information on wheat *TaTPP* genes

Gene name	Locus_ID	Chr.	Position (bp)		ORF	Introns	Deduced polypeptide			Trehalose_Ppase	
			Start	End			Length (aa)	MW (Da)	pI	Start (aa)	End (aa)
TaTPP1-1A	TraesCS1A02G210400	1A	372,639,121	372,643,307	1146	9	381	42,614.46	5.61	119	364
TaTPP1-1B	TraesCS1B02G224300	1B	402,147,526	402,150,391	1146	12	381	42,660.51	5.53	119	364
TaTPP1-1D	TraesCS1D02G213700	1D	298,692,275	298,696,295	1146	9	381	42,625.49	5.53	119	364
TaTPP2-2A	TraesCS2A02G161000	2A	111,921,839	111,925,164	1074	8	357	39,430.21	6.11	106	339
TaTPP2-2B	TraesCS2B02G187000	2B	161,722,263	161,725,704	1077	8	358	39,621.52	6.04	107	340
TaTPP2-2D	TraesCS2D02G168100	2D	111,588,533	111,591,881	1077	8	358	39,664.41	5.77	107	340
TaTPP3-2A	TraesCS2A02G161100	2A	112,744,717	112,747,766	1077	8	358	39,625.56	7.14	107	340
TaTPP3-2B	TraesCS2B02G187100	2B	162,007,594	162,010,969	1077	8	358	39,606.47	6.57	107	340
TaTPP3-2D	TraesCS2D02G168200	2D	112,099,169	112,102,442	1077	8	358	39,503.35	6.84	107	340
TaTPP4-2A	TraesCS2A02G161200	2A	113,309,228	113,312,068	1077	8	358	39,632.62	6.77	107	340
TaTPP4-2B	TraesCS2B02G187200	2B	162,445,597	162,448,929	1077	8	358	39,653.69	7.12	107	340
TaTPP4-2D	TraesCS2D02G168300	2D	112,177,996	112,181,524	1179	9	392	43,583.32	6.44	141	374
TaTPP5-2A	TraesCS2A02G167100	2A	119,307,539	119,314,162	750	8	249	28,665.56	8.28	2	230
TaTPP5-2B	TraesCS2B02G193300	2B	168,831,609	168,853,380	1680	11	559	62,281.71	8.61	312	540
TaTPP6-2A	TraesCS2A02G412100	2A	669,749,666	669,753,186	1113	9	370	41,228.87	5.70	112	347
TaTPP6-2B	TraesCS2B02G430700	2B	619,679,522	619,682,850	1143	9	380	42,099.78	5.75	112	348
TaTPP6-2D	TraesCS2D02G409300	2D	524,105,415	524,108,680	1113	7	370	41,112.88	5.58	112	347
TaTPP7-3A	TraesCS3A02G085700	3A	55,223,622	55,255,982	1662	9	553	61,554.27	8.06	306	533
TaTPP7-3D	TraesCS3D02G085800	3D	43,259,299	43,283,531	1755	9	584	65,022.10	8.86	337	564
TaTPP8-5A	TraesCS5A02G190000	5A	394,181,080	394,183,400	1122	5	373	40,852.40	8.95	114	345
TaTPP8-5B	TraesCS5B02G193100	5B	348,448,002	348,450,302	1122	4	373	40,926.44	8.97	114	345
TaTPP8-5D	TraesCS5D02G200800	5D	303,758,772	303,761,166	1122	5	373	40,860.33	8.96	114	345
TaTPP9-6A	TraesCS6A02G248400	6A	461,143,866	461,147,635	1119	8	372	41,114.13	5.68	119	355
TaTPP9-6B	TraesCS6B02G276300	6B	500,209,451	500,213,722	1119	8	372	41,281.38	5.89	119	355
TaTPP9-6D	TraesCS6D02G230500	6D	323,712,099	323,716,021	1119	8	372	41,047.03	5.56	119	355
TaTPP10-6A	TraesCS6A02G301800	6A	535,151,913	535,154,867	1251	8	416	45,367.17	9.26	114	341
TaTPP10-6B	TraesCS6B02G330900	6B	581,079,293	581,082,545	1224	8	407	44,291.96	8.64	154	381
TaTPP10-6D	TraesCS6D02G281100	6D	388,537,685	388,540,648	1110	8	369	40,252.37	8.79	114	341
TaTPP11-7A	TraesCS7A02G180800	7A	135,006,112	135,008,690	1086	9	361	39,542.13	8.41	108	335
TaTPP11-7B	TraesCS7B02G085800	7B	97,972,425	97,975,038	1095	9	364	40,160.77	8.11	108	335
TaTPP11-7D	TraesCS7D02G182600	7D	136,013,159	136,015,620	1092	9	363	39,907.51	8.60	108	335

### Phylogenetic and synteny analysis of the wheat *TPP* gene family

**Fig. 1:**
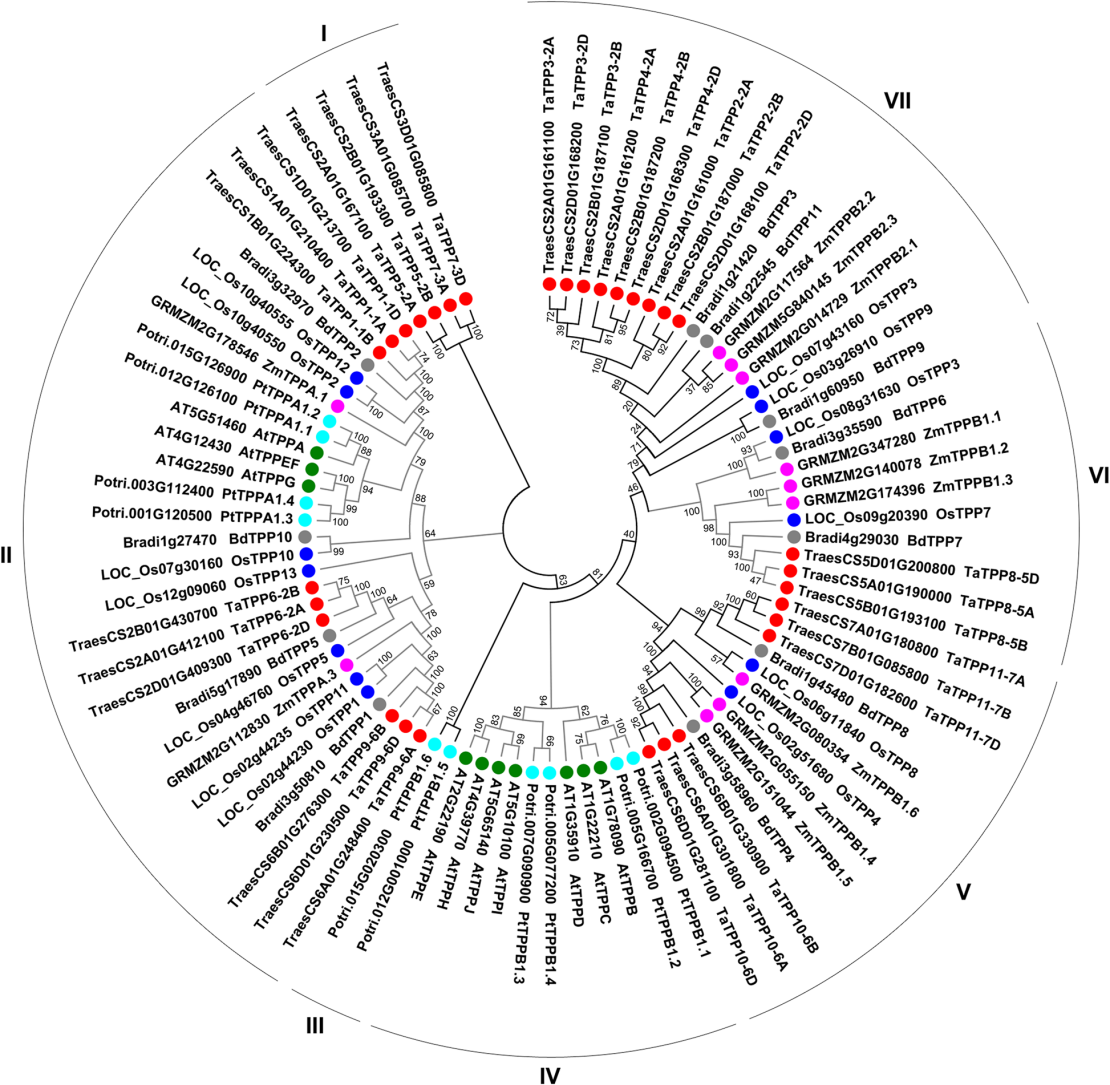
Phylogenetic relationships of the TPP family in plants. The phylogenetic tree of TPP proteins from wheat, maize, rice, *Populus*, *Arabidopsis*, and *B. distachyum*. MEGA 6.0 was used to build a neighbor-joining tree, which was visualized with the online tool Evolview-v2(https://evolgenius.info//evolview-v2/#login). TPPs from one plant species are marked with leaf labels. I-VII denotes the seven plant TPP protein subfamilies. The percentage bootstrap scores were calculated from 1000 replications

Previous study have revealed that *TPP* genes are diversified with most clades being characteristic of either monocots or eudicots [1]. To further assess the phylogenetic relationship of the *TPP* gene families in plants, 86 TPP proteins from both wheat and other plant species, including monocotyledonous angiosperms maize (*Zea mays*), *Brachypodium distachyon*, and rice (*Oryza sativa*), and the dicotyledonous angiosperms *Arabidopsis thaliana* and poplar (*Populus trichocarpa*), were used to produce a phylogenetic tree, which categorized the TPPs into six subfamilies (I-VII) (Fig. 1; Additional file 2: Fig. S1). Subfamily II was comprised of *TPP* genes from six different species, while subfamilies III and IV were comprised only of *TPP* genes from the dicotyledonous angiosperms *Arabidopsis thaliana* and poplar. Subfamilies V, VI, and VII were comprised of *TPP* genes from monocotyledonous angiosperms maize, rice, wheat, and *Brachypodium distachyon*. Analysis of the phylogenetic tree indicates the presence of 4, 9, 6, 3, and 9 TaTPPs in the TPP subfamilies I, II, V, VI, and VII, respectively (Fig. 1).

**Fig. 2:**
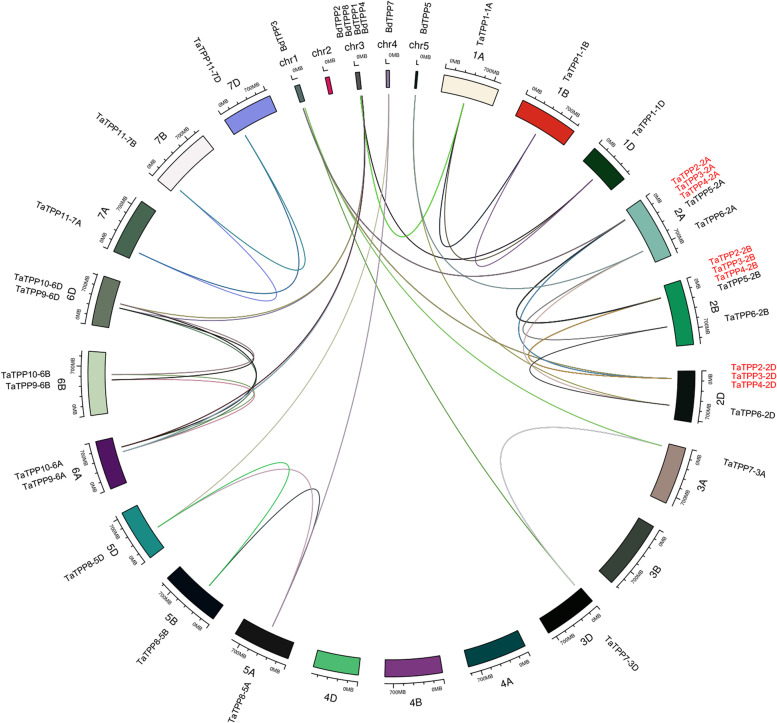
Mapping and analysis of synteny of *TaTPP* genes. The chromosome locations and syntenic relationships were visualized using circlize packages by R. *B. distachyum* and wheat chromosomes are represented as circles. The location of each *BdTPP* and *TaTPP* gene is denoted with a small black line on the circle. Colored curves indicate syntenic relationships between *B. distachyum* and wheat *TPPs* genes

Genomic comparison is a fast and easy method to transport genomic information from a well-studied species to a newly-studied species. We used the genomic position information to locate 31 *TaTPP* genes over 17 wheat chromosomes, which ranged from 1 to 5 members per chromosome (Table 1; Fig. 2). We used Holub’s method [35] to identify nine tandem duplication events (*TaTPP2-2A*/*TaTPP3-2A*, *TaTPP2-2A*/*TaTPP4-2A*, *TaTPP3-2A*/ *TaTPP4-2A*, *TaTPP2-2B*/*TaTPP3-2B*, *TaTPP2-2B*/*TaTPP4-2B*, *TaTPP3-2B*/*TaTPP4-2B*, *TaTPP2-2D*/*TaTPP3-2D*, *TaTPP2-2D*/*TaTPP4-2D*, and *TaTPP3-2D*/*TaTPP4-2D*) in wheat *TPP* genes, suggesting that certain *TaTPP* genes could be produced via gene duplication (Fig. 2). *Brachypodium distachyon* and wheat have a close phylogenetic relationship, and is considered a model for monocotyledonous angiosperm plants. As such, we performed a synteny analysis (with *E*-value <1e^− 5^) between wheat and *Brachypodium distachyon TPP* genes to explore their relationship and identified 14 pairs of syntenic *TPP* genes between *Brachypodium distachyon* and wheat, including 17 *TaTPP* genes (*TaTPP1-1A*, *TaTPP1-1D*, *TaTPP3-2A*, *TaTPP3-2D*, *TaTPP6-2A*, *TaTPP6-2D*, *TaTPP7-3A*, *TaTPP7-3D*, *TaTPP8-5A*, *TaTPP8-5D*, *TaTPP9-6A*, *TaTPP9-6D*, *TaTPP10-6A*, and *TaTPP10-6D*) and seven *BdTPP* genes (*BdTPP1*, *BdTPP2*, *BdTPP3*, *BdTPP4*, *BdTPP5*, *BdTPP7*, and *BdTPP8*) (Fig. 2). This results suggests that most *TPP* genes existed before *Brachypodium distachyon* and wheat diverged.

### Analysis of gene structure and motif composition

**Fig. 3:**
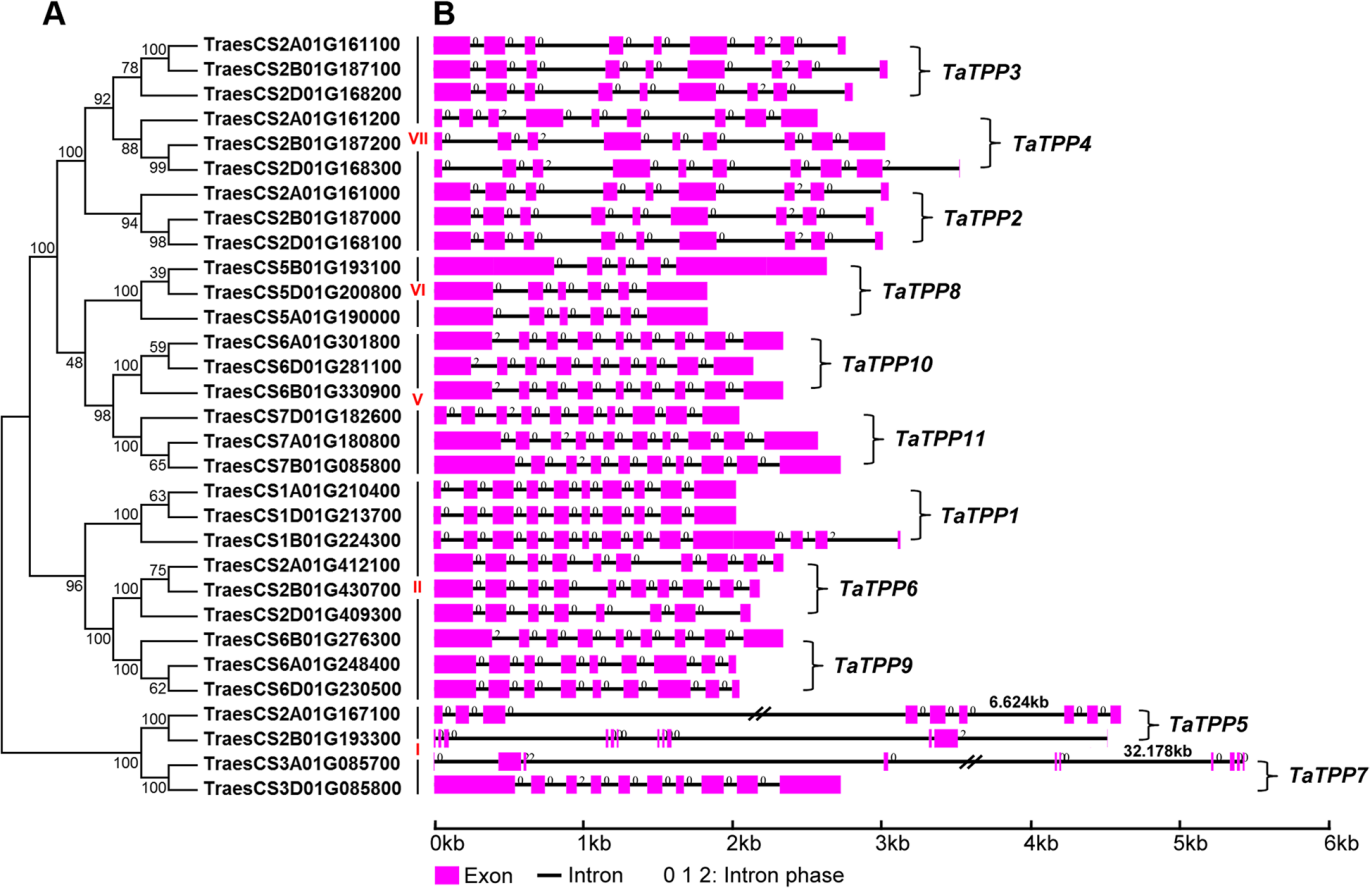
Gene structures and phylogenetic relationships of wheat *TPP* genes. **A** Phylogenetic tree of 31 full-length wheat TPP proteins generated with MEGA 6.0 and the Neighbor-Joining (NJ) method with 1000 bootstrap values. **B** Exon/intron structures of *TPP* genes in wheat. Exons and introns denoted by purple boxes and black lines, respectively

The structural divergence of exons and introns served an important role as several families of genes have evolved [36]. We generated a different phylogenetic tree using 31 full-length TaTPP protein sequences to better understand the diversity of the structure of *TaTPP* genes. The TaTPP proteins were divided into five separate subfamilies, according to the above description (Fig. 3A). Next, the locations of the exons/introns to the coding regions of each *TaTPP* gene were mapped. We found that *TaTPP8-5B* had four introns, *TaTPP8-5A* and *TaTPP8-5D* had five introns, *TaTPP6-2D* had seven introns, *TaTPP5-2B* had eleven introns, and *TaTPP1-1B* had twelve introns. Of the remaining proteins, there were 15 *TaTPP* genes had 8 introns and 10 *TaTPPs* had 9 introns (Fig. 3B). The gene structure of orthologous genes is typically highly conserved, which helps to determine their evolutionary relationships [36]. *TaTPP* genes in the same subfamily typically have similar gene structures (intron number and exon length), particularly those of subfamily VII, which all had eight introns in wheat (Fig. 3B). The hexaploid bread wheat genome was generated by the merging of the *T. urartu* (subgenome A), *Aegilops speltoides* (subgenome B), and *A. tauschii* (subgenome D) genomes hundreds of thousands of years ago. Most of the genes in the A, B, and D sub-genomes (60.1–61.3%) have orthologs in all related diploid genomes. Analysis of the related intron/exon gene structures based on the phylogenetic tree provided intron gain/loss information for all *TaTPP* genes in the A, B, and D sub-genome. Of these, four clusters altered the structure of their introns/exons, such as *TaTPP1-1A/B/D*, *TaTPP5-2A/B*, *TaTPP6-2A/B/D*, *TaTPP8-5A/B/D* (Fig. 3B). Due to the high number of orthologs in the wheat A, B, and D sub-genomes, the gain or loss of introns in these orthologs complicates the transcriptomes and proteomes found in wheat.

**Fig. 4:**
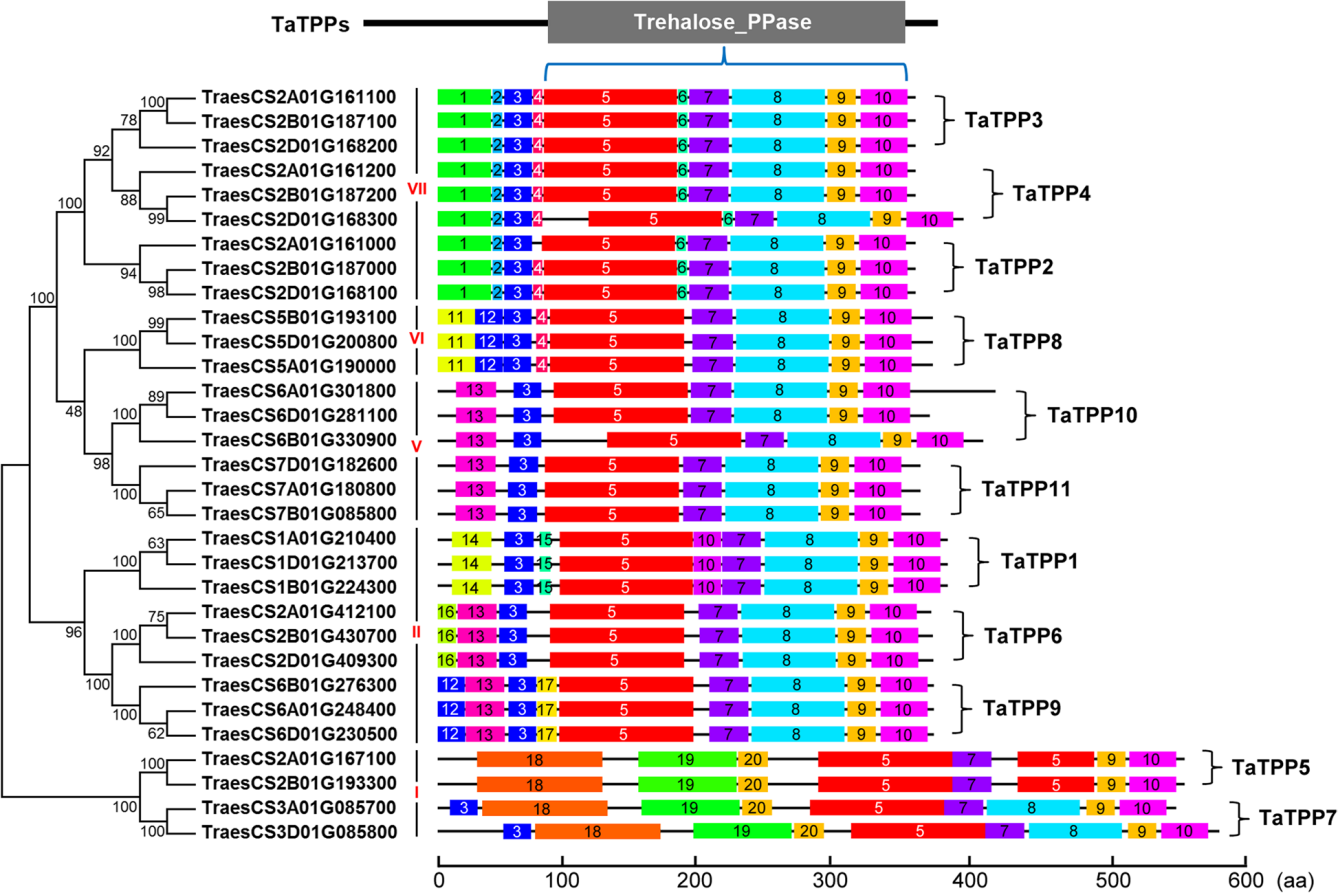
Conserved motif analysis of TPP proteins in wheat. Conserved motifs were identified using MEME (Multiple Em for Motif Elicitation) suite analysis (Version 5.3.3), and TBtools was used for graphical visualization. A colored box denotes each motif, while black lines indicate non-conserve sequences. Conserved TPP domains consist of motifs 4–10

Furthermore, we used the online MEME tool to identify the conserved motifs and assess the diverse structures of wheat TPP proteins (Fig. 4; Additional file 3: Fig. S2), and found 20 conserved protein domains (with *E*-value ≤1e^− 30^) across 31 wheat TPP proteins. The TPP domain consisted of motif 5, 6, 7, 8, 9, and 10, which is a common conserved domain located in the C-terminal of all the TaTPP proteins (Additional file 4: Fig. S3). Most motifs share orders within the same subfamily, and motifs with similar compositions shared by TaTPP proteins were clustered closely (Fig. 4). This suggests that those members of a particular group have similar functional characteristics.

### Subcellular localization of TaTPP proteins in different subfamilies

**Fig. 5:**
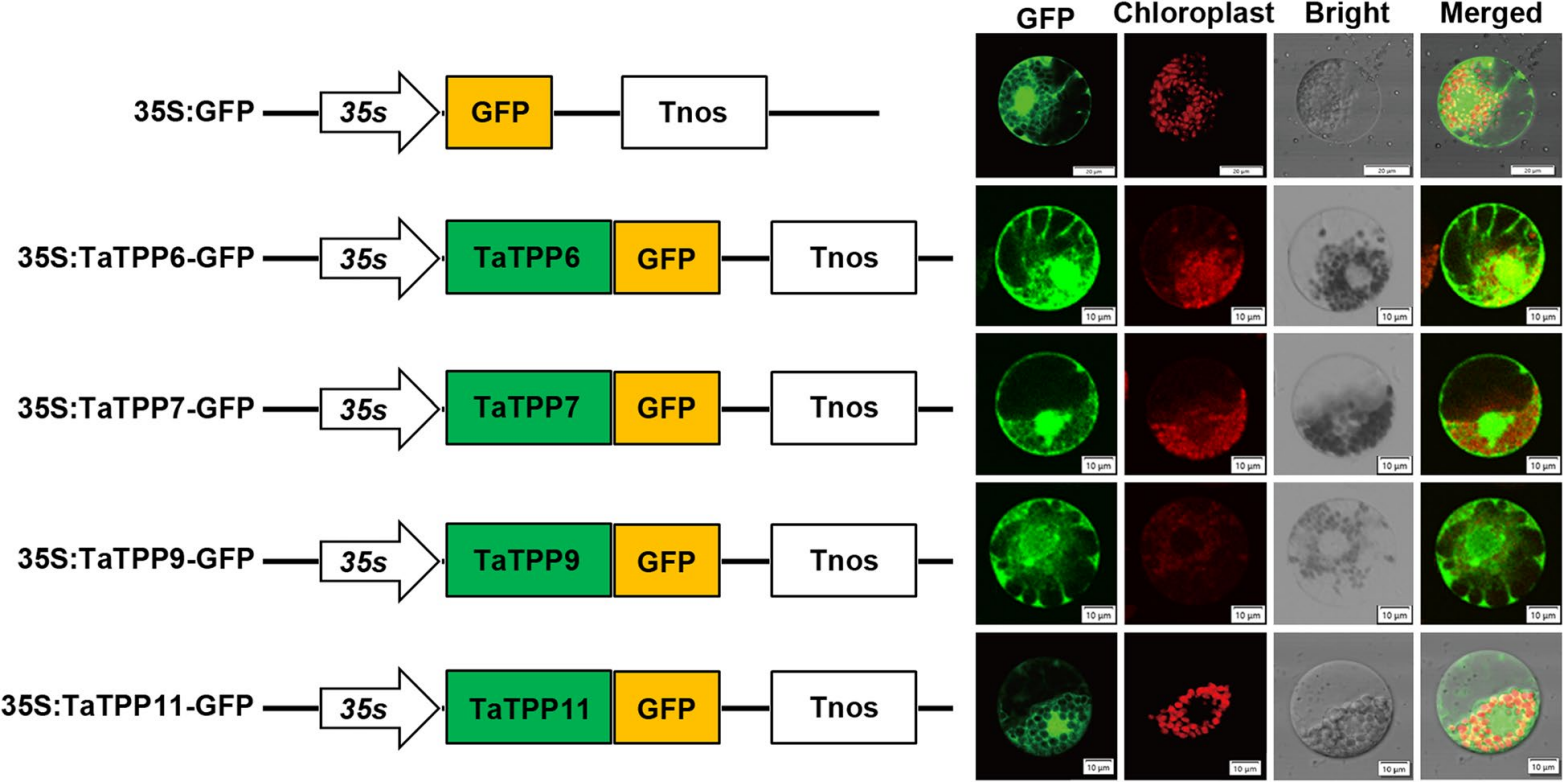
Subcellular localization of TaTPP-GFP fusion proteins in wheat mesophyll protoplasts. The 35S*::TaTPP6-GFP*, 35S*::TaTPP7-GFP*, 35S*::TaTPP9-GFP*, and 35S*::TaTPP11-GFP* fusion vectors, and 35S::*GFP* control vectors were all independently transformed into wheat mesophyll protoplasts via PEG transfection. A laser scanning confocal microscope was used to observe the green fluorescence

We further characterized the subcellular localization of four TaTPPs (TaTPP6, TaTPP7, TaTPP9, TaTPP11) that belong to the distinct cluster in the phylogenetic tree shown in Fig. 1. In order to confirm the subcellular localization of these TaTPPs, we developed the 35S::*TaTPP6-GFP*, 35S::*TaTPP7-GFP*, 35S::*TaTPP9-GFP*, and 35S::*TaTPP11-GFP* transient expression vectors to express *TaTPP6-GFP*, *TaTPP7-GFP*, *TaTPP9-GFP*, and *TaTPP11-GFP* fusion proteins in wheat protoplasts, with 35S::*GFP* as positive control. The result was as expected, all four TaTPPs-GFP fusion proteins were located in both cytoplasm and the nucleus (Fig. 5).

### *Cis*-acting regulatory elements in *TaTPP* promoters

**Fig. 6:**
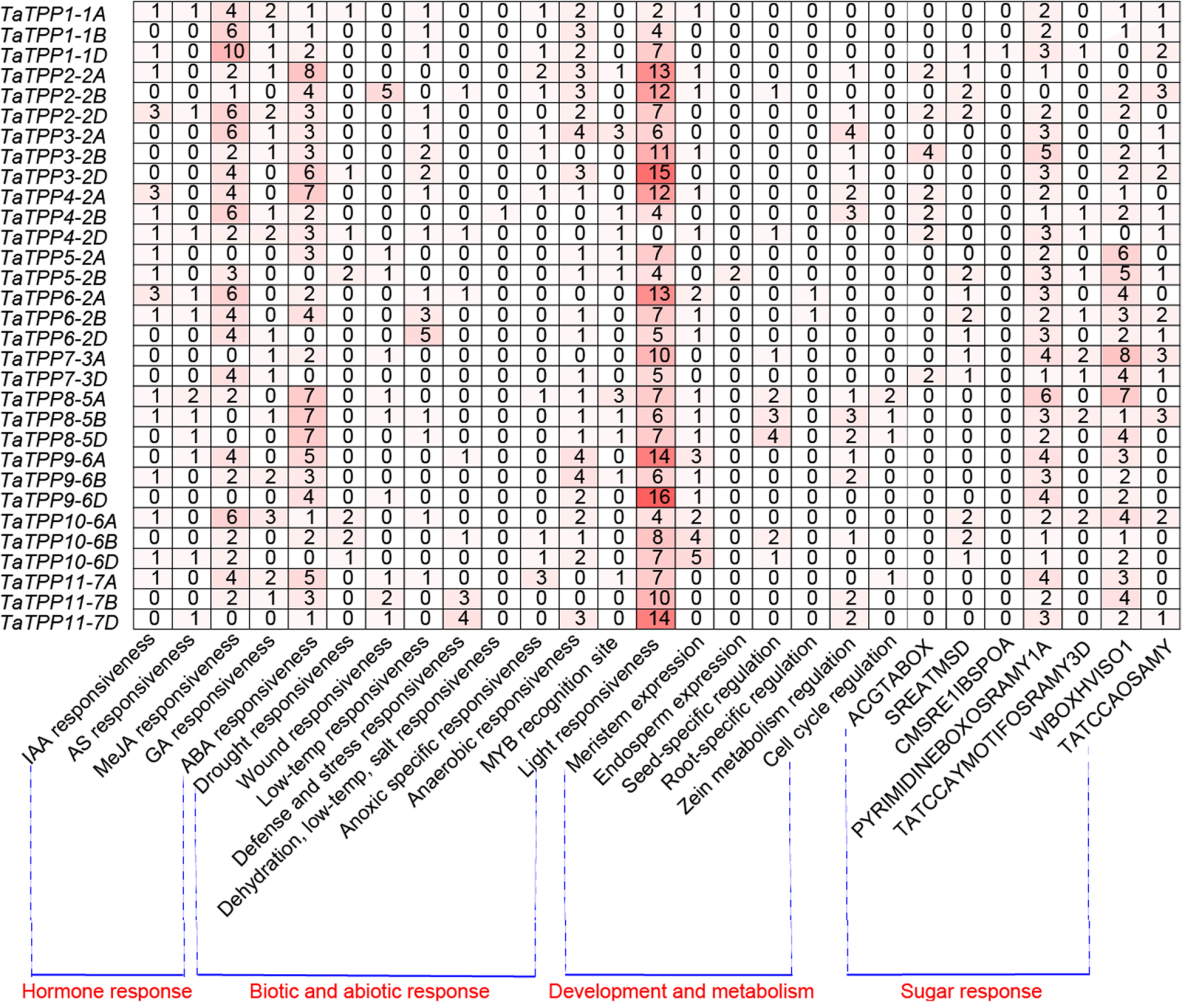
Promoter *cis*-element analysis of *TaTPP* genes. 2-kb upstream promoter sequence for all *TaTPP* genes was obtained from wheat genome database, while PlantCARE and PLACE was used to scan all the *cis*-acting regulatory elements. Numbers denote the sum of how different *cis*-acting elements respond to similar stimuli

Specific gene expression is primarily regulated by certain promoters, the action of which is mediated by transcription factors via directly binding to *cis*-acting regulatory elements [37]. Therefore, analyzing upstream regulatory sequences will contribute to a better understanding of how target genes are regulated, allowing us to assess potential functions [38]. We extracted and scanned ~ 2000 bp of non-coding sequences upstream from the predicted translation start site of each *TaTPP* gene to fully identify the putative *cis*-acting regulatory elements. Online software tools PlantCARE and PLACE were used to predict the abundant regulatory cores associated with responses to hormones, stress, sugar and development (Fig. 6; Additional file 5: Table S2).

We observed significantly enriched hormone-related motifs in the majority of the regulatory regions of the *TaTPP* genes, including abscisic acid (ABRE-element), auxin (TGA-element, AuxRE-core), gibberellin (P-box, GARE-motif and TATC-box), salicylic acid (TCA-element), and methyl jasmonate (TGACG- and CGTCA-motif). Statistical analysis indicated that two kinds of stress-related motifs are involved in abscisic acid and MeJA (methyl jasmonate), which were the most common *cis*-acting hormone-responsive elements. These elements were found in the promoters of most *TaTPP* genes, except *TaTPP5-2B*, *TaTPP6-2D*, *TaTPP7-3D* and *TaTPP10-6D* for ABA response and *TaTPP5-2A*, *TaTPP7-3A*, *TaTPP8-5B*, *TaTPP8-5D*, *TaTPP9-6D*, and *TaTPP11-7D* for MeJA response. Of the 31 *TaTPP* genes, 17 contained both gibberellin-response elements (P-box, GARE-motif and TATC-box) and auxin-response elements (TGA-element or AuxRE-core). We also found the salicylic acid-responsive TCA-element in the promoters of 11 *TaTPP* genes (Fig. 6; Additional file 5: Table S2).

Along with hormone-related elements, we observed stress elements in the *TaTPP* gene promoters. In particular, elements pertaining to light response were found in all *TaTPP* gene promoters, including G-box, TCT-motif, I-box, Sp1, and MRE. Regarding drought response, seven *TaTPP* gene promoters possessed DRE (dehydration-responsive element) or MBS (MYB binding site involved in drought-inducibility) elements. LTR is a low-temperature response element and is a primary component of the motifs related to stress observed in 16 *TaTPPs* promoters. A WUN-motif wound response element was found in 10 *TaTPP* genes, while the other seven *TaTPPs* genes possessed TC-rich repeats, which are *cis*-acting elements associated with defense and stress responses. Besides, we observed sugar-responsive elements in the *TaTPP* gene promoters, Seven sugar response related elements were identified in the promoter region of *TaTPPs* through PLACE online software. Among them, only the *TaTPP1-2D* promoter region has CMSRE1 (Carbohydrate Metabolite Signal Responsive Element 1) elements. Except for *TaTPP2-2B*, the remaining TPPs have PYRIMIDINEBOXOSRAMY1A (pyrimidine box are partially involved in sugar repression) element; ACGTABOX elements was predicted in promoters of *TaTPP2-2A*, *TaTPP2-2D*, *TaTPP2–3-2B*, *TaTPP4-2A/B/D*, *TaTPP7-3D;* WBOXHVISO1 appear in the promoter region of TPPs except *TaTPP4-2D*, *TaTPP3-2A*, *TaTPP2-2A.* A large number of sugar response elements have been identified in the *TPP* promoter region, indicating that *TaTPPs* were likely to be relevant for a sugar-regulated pathway. Certain *cis*-elements are involved in the specific expression in organs and tissues or with metabolism, including the role of MBS I in flavonoid biosynthetic genes regulation, the role of motif I in root-specific expression, the role of CAT-box in meristem expression, the role of GCN4 motif in endosperm expression, the role of the RY-element in seed-specific regulation, and the role of O_2_-site in zein metabolism regulation, and the role of MSA-like in cell cycle regulation (Fig. 6; Additional file 5: Table S2). These results indicated that *TaTPP* genes might be involved in plant development, multiple hormone and stress responses.

### Tissue-specific expression profiles of *TaTPP* genes

**Fig. 7:**
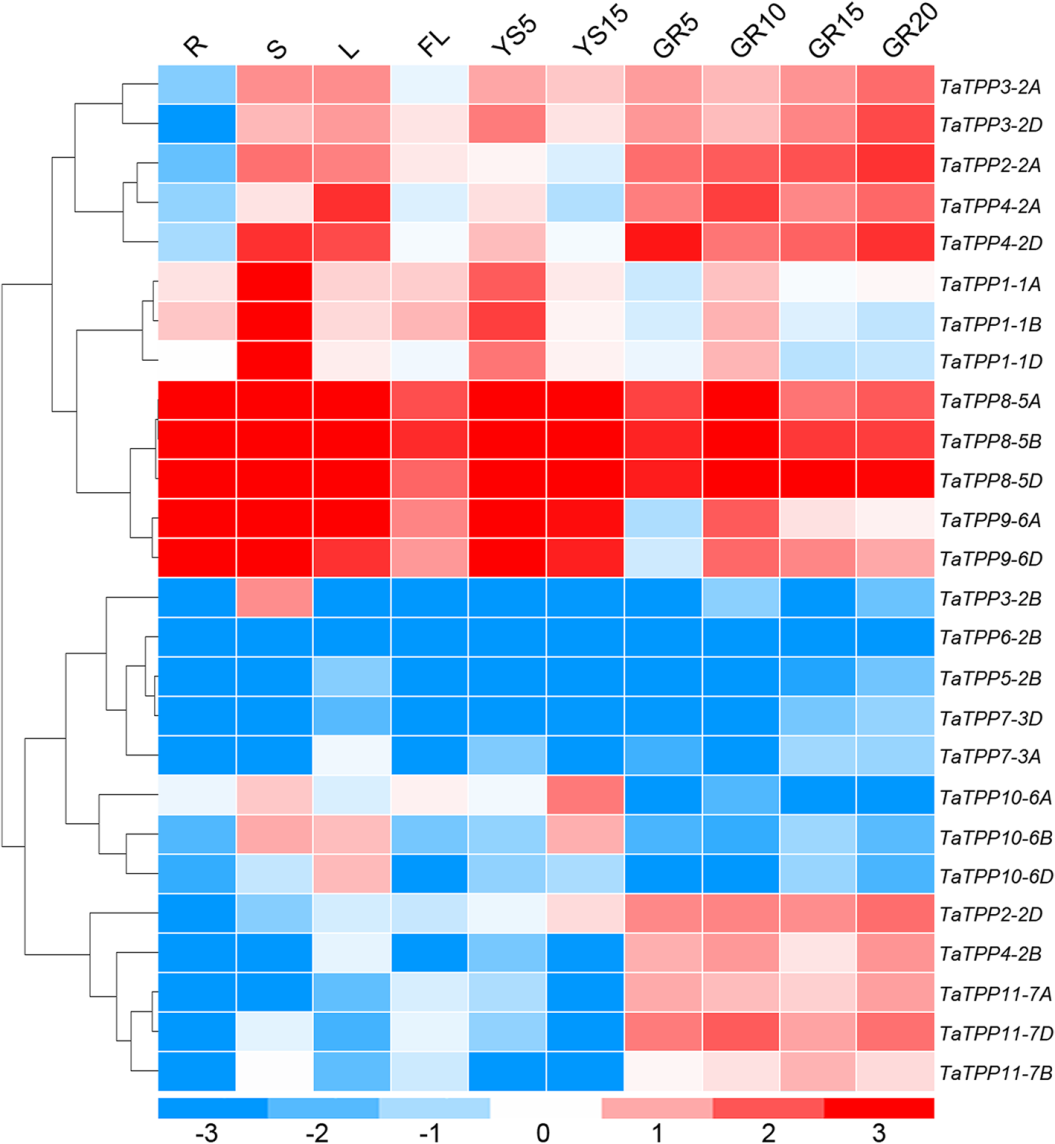
*TaTPP* gene expression profiles in ten different organs or tissues. Heatmap drawn from Log_10_-transformed expression values. Blue or red indicates the lower or higher levels of expression of each transcript in each sample, respectively. R, root of wheat seedling at five-leaf stage; S, stem of wheat seedling at five-leaf stage; L, leaf of wheat seedling at five-leaf stage; FL, flag leaf at heading stage; YS5, young spike at early booting stage; YS15, spike at heading stage; GR5, grain of 5 days post-anthesis; GR10, grain of 10 days post-anthesis; GR15, grain of 15 days post-anthesis; GR20, grain of 20 days post-anthesis

Gene expression is required for the normal growth and development of plants. Specific patterns of expression of candidate genes indicate potential roles in both growth and development. We used publicly available RNA-seq data to observe these expression patterns in seedling stems, seedling roots, seedling leaves, flag leaves, and during two stages of spike development (5 days and 15 days after head sprouting) and four stages of grain development (5 days, 10 days, 15 days, and 20 days after pollination), allowing us to assess the possible role of *TaTPP* genes during the growth and development of wheat. We obtained 26 *TaTPP* gene transcripts (Fig. 7), and could not locate five other *TaTPP* genes due to low levels of expression or the fact that they could be pseudogenes. Levels of expression vary widely in different tissues of wheat *TaTPP* genes, and between different tissues in individual *TaTPP* genes. We observed three homologous genes *TaTPP8-5A/B/D* that demonstrated widespread expression patterns that were higher in almost all tissues and stages. There are high levels of *TaTPP1-1A/B/D* expression in both seedling stems and young spikes, while there are high levels of *TaTPP2-2A* and *TaTPP4-2A/D* expression in seedling leaves, seedling stems, and grains. Compared with the seedling roots, *TaTPP3-2A/D* display relatively higher expression in other tissues and stages. There are high levels of *TaTPP9-6A/D* expression in seedling leaves, roots, spikes, and stems. There are higher levels of *TaTPP10-6A/D* expression in seedling stems, leaves, and mature spikes, while there is a strong and particular expression of *TaTPP2-2D*, *TaTPP4-2B*, and *TaTPP11-7A/B/D* during grain development, suggesting that these genes could play significant roles during this stage (Fig. 7).

Most homologous genes demonstrate similar patterns of expression during developmental stages, though several clustered expression profiles do not have similar genes, including copies of individual kinds of *TaTPP* genes from their sub-genomes; some *TaTPP* homologous genes demonstrate opposite expression patterns. For example, *TaTPP2-2A* was found on chromosome 2A and was preferentially expressed in the seedling leaves and stems, while the homologous *TaTPP2-2D* gene (located on chromosome 2D) was expressed in these tissues at a lower point. *TaTPP10-6A* was located on 6A and displays higher levels of expression in mature seedling stems and spikes. The homologous *TaTPP10-6B*, found on 6B, was expressed preferentially in the seedling leaves, stems, and mature spikes, while homologous genes from 6D was expressed only in the seedling leaves (Fig. 7). This difference in expression profiles between homologous genes from different subgenomes demonstrated that some *TaTPPs* had acquired new functions or lost old functions following polyploidization during wheat’s evolutionary history.

### Expression analysis of *TaTPP* genes respond to abiotic stresses

**Fig. 8:**
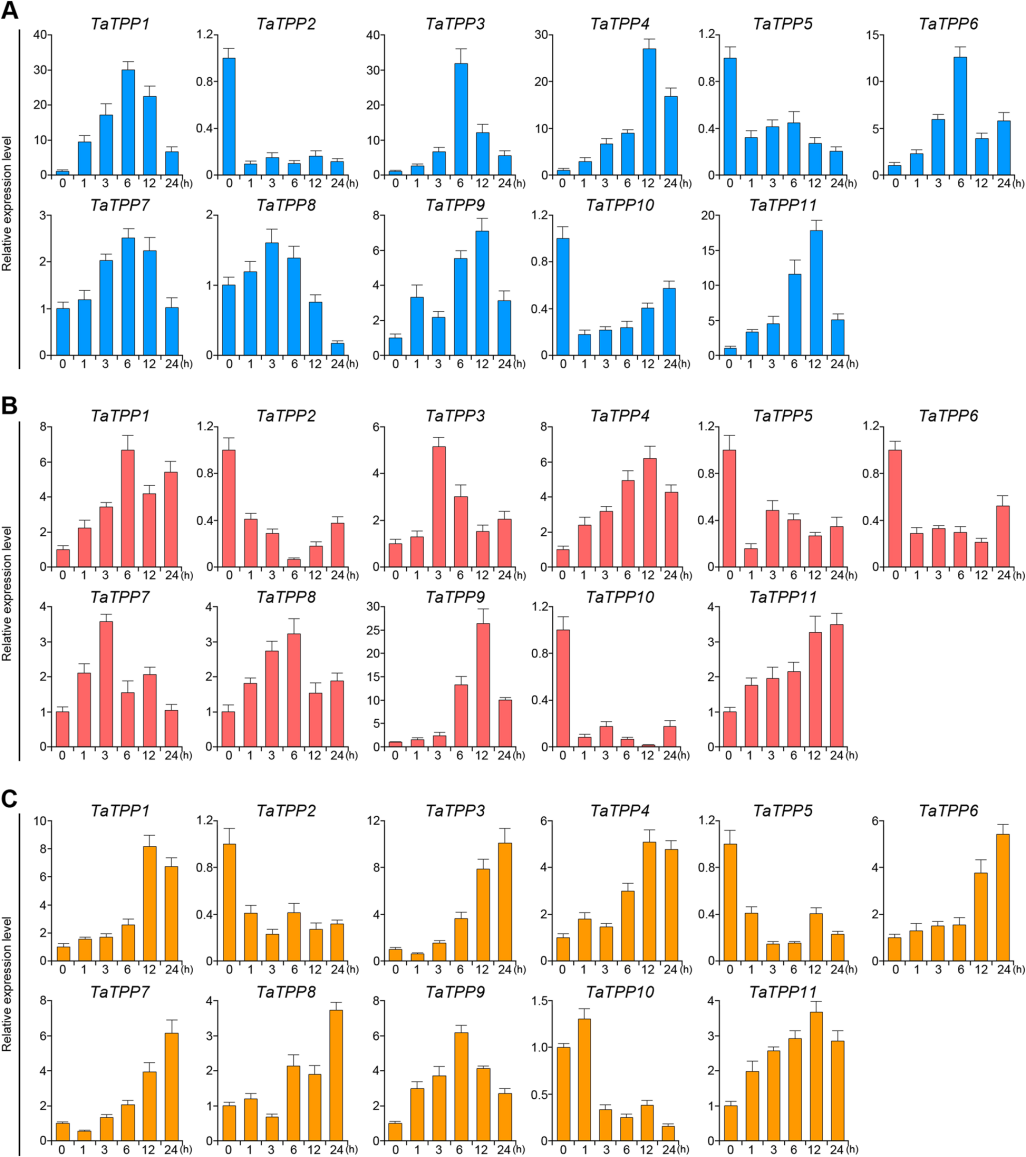
*TaTPP* gene expression profiles responding to abiotic stresses. **A** *TaTPP* gene expression patterns after ABA treatment. **B** *TaTPP* gene expression pattern under low-temperature treatment. **C** *TaTPP* gene expression pattern under salt stress treatment

Environmental stresses significantly affect the productivity of wheat, making it important to study the wheat genes responsible for stress response in order to increase yields. We used quantitative real-time PCR (qRT-PCR) to assess how *TaTPP* gene expression responds to continuous ABA, low temperature, and salt stress, allowing us to analyze the role of *TaTPP* genes that could be associated with plant defense to abiotic stresses. We designed allele pairs from A-, B- and D-subgenomes and tested them together, as the products of their transcription share similar sequences. Each gene we analyzed had a different expression when responding to at least one abiotic stress (Fig. 8). In response to ABA, there were eight up-regulated *TaTPPs* (*TaTPP1*, *TaTPP3*, *TaTPP4*, *TaTPP6*, *TaTPP7*, *TaTPP8*, *TaTPP9*, and *TaTPP11*) and three down-regulated *TaTPPs* (*TaTPP2*, *TaTPP5*, and *TaTPP10*) in seedling leaves at a minimum of one time point. As for response to low-temperature conditions, there were seven up-regulated *TaTPPs* (*TaTPP1*, *TaTPP3*, *TaTPP4*, *TaTPP7*, *TaTPP8*, *TaTPP9*, and *TaTPP11*) and four down-regulated *TaTPPs* (*TaTPP2*, *TaTPP5*, *TaTPP6*, and *TaTPP10*). In addition, there were eight up-regulated *TaTPPs* (*TaTPP1*, *TaTPP3*, *TaTPP4*, *TaTPP6*, *TaTPP7*, *TaTPP8*, *TaTPP9*, and *TaTPP11*) and three down-regulated *TaTPPs* (*TaTPP2*, *TaTPP5*, and *TaTPP10*) were responsive to salt stress (Fig. 8).

**Fig. 9:**
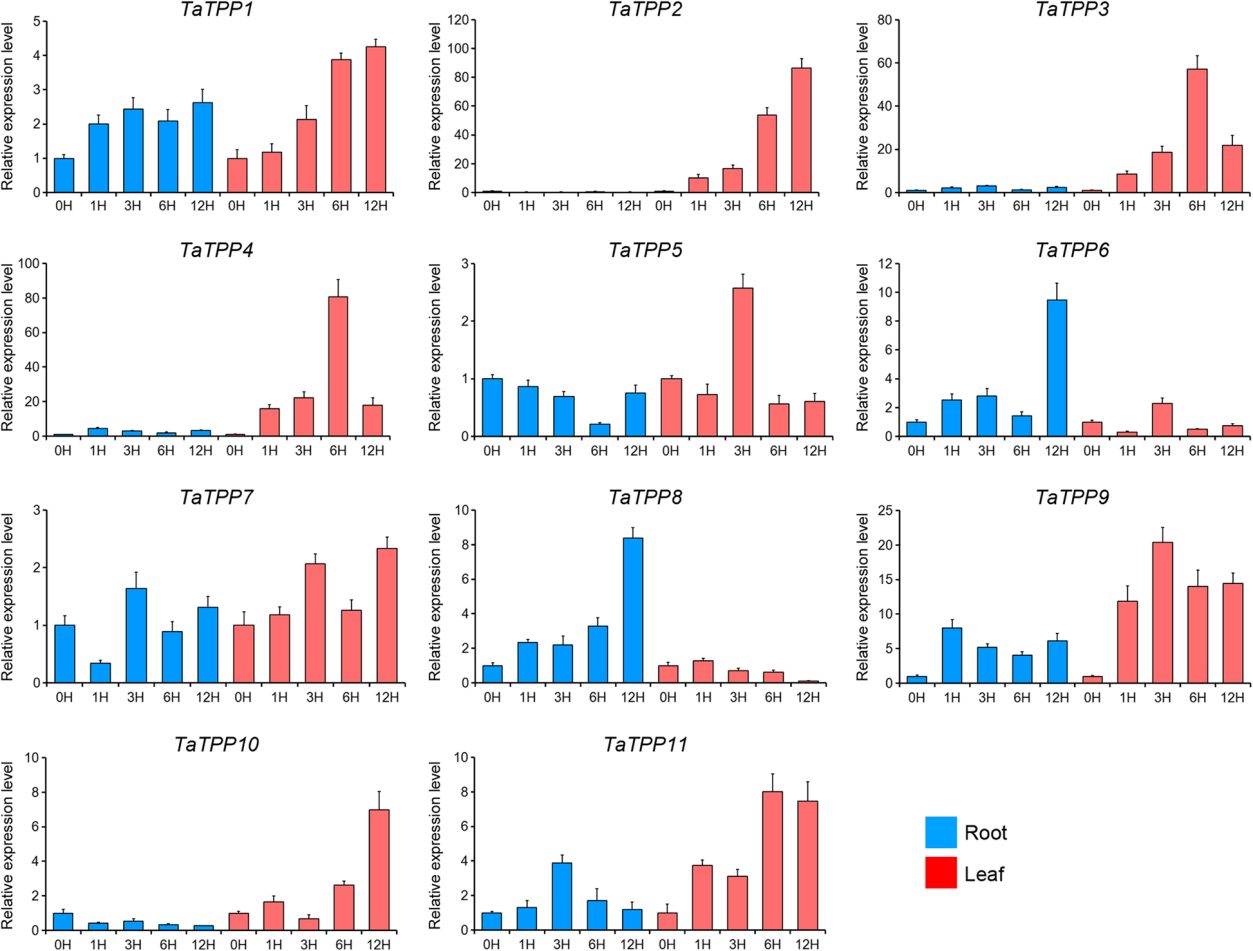
*TaTPP* gene expression profile in seedling roots and leaves under drought stress conditions. Red and blue columns represent level of *TaTPP* gene expression under 20% PEG treatment in plant leaves and roots obtained from wheat seedlings, respectively. X-axis indicates time points following drought treatment. Expression data from the control sample were normalized to 1, while error bars indicate standard error from three replicates

To better understand the functions of *TaTPP* genes in regulating wheat drought response, the expression patterns of 11 *TaTPPs* were experimentally examined in leaves and roots of 3-week-old drought-treated wheat seedlings. As illustrated in Fig. 9, a dramatic upregulation of 8 *TaTPP* genes (*TaTPP1*, *TaTPP2*, *TaTPP3*, *TaTPP4*, *TaTPP5*, *TaTPP9*, *TaTPP10*, and *TaTPP11*) were observed in response to drought stress, especially in the leaves. *TaTPP7* also showed a slightly up-regulation in leaves and roots after drought stress. The changed expression levels of *TaTPP2*, *TaTPP3* and *TaTPP4* in leaves after drought stress were very sharp, with more than 60 folds, indicating that these genes are extremely susceptible to drought stress. Some genes showed very similar expression profiles after drought stress, such as *TaTPP3* and *TaTPP4* pairs. Some *TaTPP*s were significantly upregulated after light/early drought stress, such as *TaTPP2*, *TaTPP3*, *TaTPP4* and *TaTPP9* in leaves and *TaTPP9* and *TaTPP11* in roots, suggesting positive roles of these genes in early drought stress response. Some genes were significantly upregulated after severe stress, such as *TaTPP1*, *TaTPP10* and *TaTPP11* in leaves and *TaTPP6* and *TaTPP8* in roots, suggesting that these genes are important for the plant response to drought stress at a severe level. Upon drought stress, *TaTPP5* and *TaTPP10* were upregulated in leaves but downregulated in roots (Fig. 9). These data show the potential of some *TaTPP* genes for enhancing adversity resistant capacity, especially for wheat drought improvement.

### Ectopic expression of *TaTPP11* in *Arabidopsis* delayed plant development

**Fig. 10:**
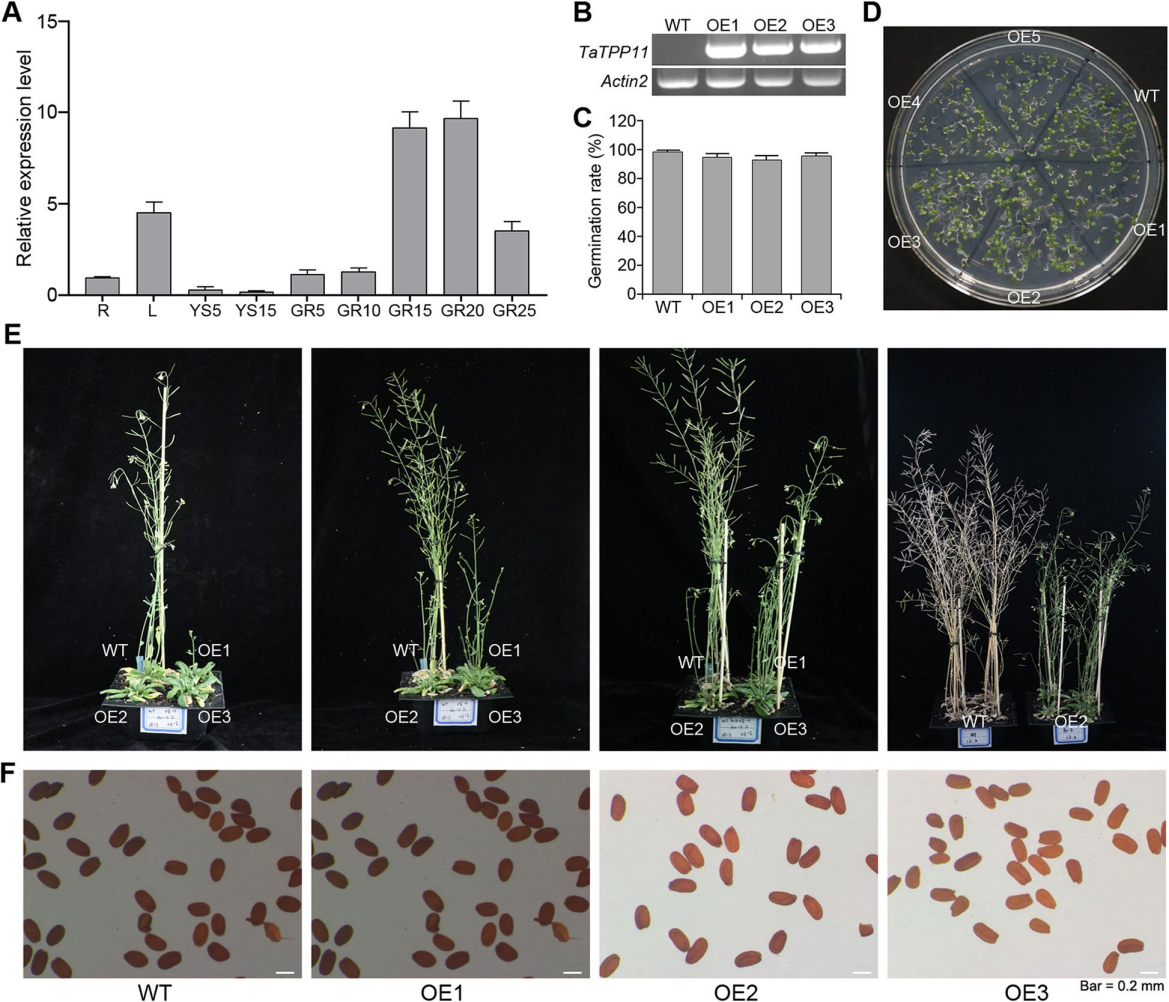
Comparing plant morphology between WT plants and *TaTPP11* overexpression. **A** *TaTPP11* expression profiles in various tissues. R, root of wheat seedling at five-leaf stage; L, leaf of wheat seedling at five-leaf stage; YS5, young spikes of 5 days after booting; YS15, spikes 15 days after booting; GR5, grain of 5 days post-anthesis; GR10, grain of 10 days post-anthesis; GR15, grain of 15 days post-anthesis; GR20, grain of 20 days post-anthesis; GR25, grain of 25 days post-anthesis. **B** RT-PCR identification of *Arabidopsis* lines overexpressing *TaTPP11-7D*. **C-D** Seed germination assay between *35S:TaTPP11-7D* transgenic and WT plants. **E-F** Plant phenotype (**E**) and seed morphology (**F**) of wild-type and *35S:TaTPP11-7D* transgenic *Arabidopsis* plants

Alignment of the protein sequences determined the presence of three *TaTPP11* homeologs sharing a sequence similarity of approximately 95% (Additional file 6: Fig. S4). Additional information regarding the spatiotemporal profile of *TaTPP11* expression could contribute to a better understanding of how *TaTPP11* functions biologically. In this case, we observed *TaTPP11* expression across various tissues and organs of wheat at different stages of development, such as the roots and leaves of seedlings, young panicles, flag leaves, and seeds. Our results demonstrated high levels of *TaTPP11* expression in seedling leaves and developing seeds, and low levels of *TaTPP11* expression in developing panicles (Fig. 10A). This indicates that *TaTPP11* could serve an important purpose as wheat seeds develop.

To better understand how *TaTPP11* regulates to the development of wheat, we produced 35S*::TaTPP11-7D* transgenic *Arabidopsis* lines and assessed their levels of *TaTPP11-7D* expression to select three independent transgenic lines (*OE1*, *OE2*, and *OE3*) for subsequent analysis (Fig. 10B; Additional file 7: Fig. S5). Firstly, the germination of the seeds were observed that there were no significant differences between transgenic and wild type lines (Fig. 10C, D). Next, we detected the phenotypes of the 35S*::TaTPP11-7D* transgenic *Arabidopsis* lines and that of the wild-type throughout the developmental stages. The *TaTPP11-7D* transgenic *Arabidopsis* seedlings grew vegetation for much longer, bolted and flowered later, and had a lower plant height compared to the wild type (Fig. 10E). We also analyzed the organs from both the *Arabidopsis* wild-type plants and transgenic lines, and no significant differences in the organs were found between the transgenic and wild type varieties, including in the seeds, flowers, and siliques (Fig. 10E, F).

## Discussion

There is a significant body of evidence indicating that T6P is an important signal metabolite in plants that affects metabolism, growth, and development. Therefore, there is a pressing need to better understand the processes and function of several plant TPP proteins. Whole-genome sequencing and the widespread nature of worldwide genomic databases have allowed researchers to closely analyze complex genomes, such as wheat [38]. Identifying *TPP* genes in wheat is needed to additionally characterize these genes. While the family of *TPP* genes has been studied in both monocots and dicots, their specific functions are still unclear, particularly in wheat. To better understand this function, we comprehensively identified and analyzed *TPP* genes in wheat.

We identified 31 candidate genes in the family of TPP genes in wheat (Table 1). There were three times as many *TPPs* in wheat as in *Brachypodium distachyon* and rice, which is evidenced by the fact that hexaploid wheat descended from a hybridization of A-, B-, and D-genome ancestors approximately one or two million years ago [39, 40]. This study assessed certain traits found in wheat *TPP* genes. The majority of *TaTPP* genes encode proteins with a predicted molecular mass of 39–65 kDa (with the exception of TaTPP5-2A) as well as an isoelectric point of approximately 5.5–9.2, which was similar to the TPPs found in other species of plants, while the majority of *TaTPP* genes possessed 8–9 introns (Table 1). We performed multiple sequence alignment and analyzed the phylogenetics of the 31 TaTPP protein sequences. We observed significant sequence divergence, particularly at the C-terminus, during the multiple sequence alignment of wheat TaTPP proteins (Additional file 4: Fig. S3). This protein sequence diversity demonstrates that *TaTPP* genes may have multifarious roles in plant growth, development and stress response [2, 33]. We classified the wheat TaTPP protein family into five subfamilies (I, II, V, VI, VII) by assessing how they relate to homologous TPPs in other species (Fig. 1; Fig. 3A), gene structures (Fig. 3), and motif arrangements (Fig. 4). The smallest subfamily was the TaTPP VI subfamily (Fig. 1; Fig. 3A). Analysis of the gene structure demonstrated that genes from each TaTPP subfamily have similar numbers and positions of their exon-intron structures (Fig. 3B), though the TaTPP I subfamily was more divergent (Fig. 3B), which suggested that the TaTPP I subfamily genes could perform different roles during the development of wheat. Additionally, wheat TaTPP proteins all possess particular conserved TPP domains, consisting of six conserved motifs (Fig. 4). With the exception of TPP domain-based conserved motifs, there are specific motifs for each TaTPP subfamily. For example, motifs 1 and 2 exist only in TaTPP VII subfamily proteins, while motif 11 only exists in the TaTPP VI subfamily. Motifs 18, 19, and 20 only exist in the TATPP I subfamily (Fig. 4).

Duplicate genes located on different chromosomes are considered segregation duplication events. It is common for gene duplication events to occur in wheat, which will assist in the future analysis of the function and evolution of genes [34]. Whole genome duplications are common in angiosperms [41], and typically expand the gene family [42]. Gene duplication can result in the emergence of novel functions for certain plant genes. Syntenic relations and segregation duplication events between *Brachypodium distachyon* and wheat suggested that certain *TaTPP* genes were produced via gene duplication, indicating their similar origins (Fig. 2).

We analyzed the expression of publicly available RNA-seq data from 10 organs/tissues at various stages of development to assess the role play by *TPP* genes across wheat’s life cycle. Wheat *TaTPP* genes are similar to *Arabidopsis* in that they are differentially transcribed, depending on the tissue, stage, and cell [26]. High levels of *TaTPP* genes expression were detected in the leaves, such as *TaTPP2-2A*, *TaTPP3-2A/D*, *TaTPP4-2A/D*, *TaTPP8-5A/B/D*, *TaTPP9-6A/D* and *TaTPP10-6B/D* (Fig. 7). This results was similar to previous results, where higher levels of expression of *TPPA*, *TPPB*, and *TPPG* in the leaves compared to other organs [26]. There were higher levels of *TaTPP9-6A/D* and *TaTPP8-5A/B/D* expression in the roots, which is similar to *TPPA*, while there were higher levels of *TPPD*, *TPPG*, and *TPPI* in the root caps and protoderms [26], indicating that they could be involved in the development of roots. In wheat, *TaTPP2-2A/D*, *TaTPP3-2A/D*, *TaTPP4-2A/B/D*, *TaTPP8-5A/B/D*, and *TaTPP11-7A/B/D* all demonstrated higher levels of expression in the grains at various stages of development, indicating they could be involved in grain development (Fig. 7). These results indicated that *TaTPP* genes could serve a variety of roles during the development of wheat. Additional ectopic *TaTPP11* expression in *Arabidopsis* displayed delays in development and flowering (Fig. 10), highlighting the importance of *TaTPP11* during plant development. Notably, previous studies have showed that overexpression of *TPP* can promote growth and varying severity of morphological abnormalities in several species [12, 28, 29, 32, 43]. For example, overexpressing of *AtTPPB*, *AtTPPC*, *AtTPPI*, and *JcTPPJ* can result in relative short perianth and late-flowering phenotype of transgenic *Arabidopsis* [43]. In maize, *RAMOSA3* (*RA3*) encodes a TPP enzyme that controls maize inflorescence architecture by mediating the axillary meristems via modulation of trehalose and T6P levels [12, 33]. These reports collectively indicated that disruption of trehalose metabolism can cause pleiotropic effects, including leaf and inflorescence morphogenesis, and the transition from vegetative growth to flowering.

The expression or transcription of genes begins with an upstream regulatory promoter region, which is the combination of several *cis*-acting regulatory components joined with a minimal basic start element. Different regulatory cores provide the promoters with strength, time-space specificity, and stimuli response. Thus, analyzing a target gene promoter’s regulatory elements can allow us to predict how its expression will respond to different stimulation. A review of the promoters of the *TaTPP* gene family demonstrated that several phytohormone-, stress- and development-related regulatory elements were present (Fig. 6; Additional file 5: Table S2). Significant amounts of hormone-related *cis*-acting regulatory elements were observed in most *TaTPP* promoters suggest that they play important roles as regulators of the core in various hormone-signaling pathways. Members of the *TaTPP* gene family play roles in response to biotic and abiotic stresses, which is consistent with enriching several *cis*-elements related to stress. Certain *TaTPP* promoters possess regulatory cores related to stress, such as ABA-responsive elements ABRE, SA-responsive elements (TCA-elements), defense and stress-responsive elements (TC-rich repeats and MBS), low-temperature-related elements (LTR), MeJA-responsive elements (CGTCA- and TGACG-motif), and wound-responsive elements (WUN-motif) (Additional file 5: Table S2). Among them, CGTCA- and TGACG-motifs are typical MeJA-responsive *cis*-acting elements, and were found in 20 of 31 promoters of the *TaTPP* genes. Jasmonic acid, a primary growth hormone, regulates resistance to plant diseases and responses to abiotic stresses [44]. We also observed certain elements specific to organs or tissues related to the development of roots, seeds, or endosperms (Fig. 6; Additional file 5: Table S2). Promoters of *TaTPP2-2B*, *TaTPP4-2D*, *TaTPP7-3A*, *TaTPP8-5A*, *TaTPP8-5B*, and *TaTPP10-6B* possess RY-element, a seed-specific regulation core, which indicates that they could play a role in seed development. The *TaTPP6-2A* and *TaTPP6-2B* genes should be further studied, since they possess the root-specific motif I element and could play a role in the development of roots (Fig. 6; Additional file 5: Table S2).

Abiotic stresses are primarily responsible for reducing crop quality and yield. Recent researches have sought to better understand how plants respond to abiotic stresses, which would contribute to improved crop tolerance. While the overexpression of *E. coli. TPS* and *TPP* fusion proteins can increase the tolerance of abiotic stress in rice [3], there is little known about how endogenous *TPP* functions in plants. We analyzed the *cis*-acting regulatory elements and found high levels of regulatory cores in the *TaTPP* gene family promoters. We performed qRT-PCR analysis to observe how *TaTPP* genes found in the seedling leaves respond to salt, low-temperature, ABA, and drought stresses (Fig. 8; Fig. 9). Our results demonstrated that the majority of *TaTPP* genes were up-regulated after ABA treatment, while some decreased. We saw the same expression profiles after drought, salt, and low-temperature treatments. However, the intensity of induction and the whether the expression was suppressed or up-regulated was different between all stressors. All four abiotic stresses regulated *TaTPP* gene expression positively and negatively in wheat (Fig. 8; Fig. 9), though the leaves and roots responded differently to drought stress conditions (Fig. 9). The fact that *TaTPP2*, *TaTPP5*, and *TaTPP10* were all down-regulated under low-temperature, ABA, and salt stress treatments suggests they are suitable candidates for using CRISPR-Cas9 gene editing to improve the abiotic tolerance of wheat plants. *TaTPP* genes respond to stress in a manner, indicating they assist in adapting to variable environmental conditions.

## Conclusions

In this study, we identified 31 *TPP* family genes in wheat, all of which have at least one conserved TPP domain. The TPP family in wheat can be classified into five subfamilies based on their similar exon/intron structures and motifs. Additionally, there are four TaTPP protein found in the cytoplasm and nucleus (TaTPP6, TaTPP7, TaTPP9, and TaTPP11). A pattern analysis of tissue-specific expression demonstrated that *TaTPP* genes in wheat were expressed differentially, indicating that they play different roles in the growth and development of wheat. Analysis of the promoter *cis*-elements and expression patterns when subjected to abiotic and biotic stresses demonstrated that *TaTPP*s respond to different stimuli in wheat. Additionally, *TaTPP11* overexpression in *Arabidopsis* exhibits a developmentally delayed phenotype, highlighting *TaTPP11* appear to have a functional role in the regulation of development of plant. This study provides significant information on the wheat *TPP* gene family, which will allow for the future study of their functional divergence and how they can be manipulated in the future.

## Methods

### Stress treatment and plant material

We used the wheat cultivar *Chinese spring* for this study, which was acquired from Northwest A&F University. However, we also could have obtained this variety from Chinese Crop Germplasm Resources Information System (http://www.cgris.net/zhongzhidinggou/index.php). The cultivar was surface-sterilized using 75% ethanol, after which it was washed using deionized water and germinated on wet filter paper for 3 days at 25 °C. We then placed the germinated seeds in a nutrient solution (0.1 mM KCl, 0.75 mM K_2_SO_4_, 0.65 mM MgSO_4_, 0.25 mM KH_2_PO_4_, 1.0 mM MnSO_4_, 1.0 mM ZnSO_4_, 0.1 mM EDTA-Fe, 2.0 mM Ca(NO_3_)_2_, 0.005 mM (NH_4_)_6_Mo_7_O_24_, 0.1 mM CuSO_4_) and hydroponically cultivated them in a 16 °C growth chamber under a 16/8 h light/dark cycle.

For ABA and salt treatments, we immersed seedlings at the three-leaf stage into hydroponic solutions with 200 mM NaCl and 100 μM ABA, and obtained samples at 0, 1, 3, 6, 12, and 24 h after treatment. For low-temperature treatments, we immersed seedlings at the three-leaf stage into hydroponic solutions at 4 °C for 0, 1, 3, 6, 12, and 24 h and obtained samples. For drought treatment, we placed seedlings at the three-leaf stage onto a clean bench and subjected them to drought conditions (25 °C, relative humidity 40–60%) and collected the roots and leaves from three seedlings at 0, 1, 3, 6, and 12 h. We quickly froze all samples in liquid nitrogen and stored them at − 80 °C to isolate the RNA.

### Quantitative real-time PCR and RNA extraction

We used a Total RNA Rapid Extraction Kit for Polysaccharides Polyphenol Plant (BioTeke), according to the instructions of the manufacturer, to isolate and purify the total RNA. We then treated the resulting purified RNA with RNase-free DNase I (TaKaRa, China) to remove traces of DNA and ensure the sample was not contaminated. We then synthesized first-strand cDNA from 1 μg of total RNA with Recombinant M-MLV reverse transcriptase (Promega, USA) and used an ABI7300 Thermo-cycler (Applied Biosystems, USA) to conduct quantitative real time-PCR (qRT-PCR) in optical 96-well plates. All reactions were performed in 10 μl volume, with 1 μl diluted cDNA, 200 nM gene-specific primers, and 5 μl SYBR Premix Ex Taq II (TaKaRa) according to the following: 10 min at 95 °C, and then 40 cycles of 15 s at 95 °C and 30 s at 60 °C. We verified the specificity of each primer’s amplicon via melting curve analysis and used the wheat *Actin* (Gene ID: 542814) as an internal control for analyzing the expression of *TaTPP11* in wheat. We calculated relative levels of gene expression levels using the 2^−ΔΔCt^ method [45], while expression variation was estimated from three biological replicates. Additional file 8: Table S3 outlines the primer pairs used in qRT-PCR analysis.

### Genome-wide identification and annotation of *TPP* genes in wheat

Twenty-three TPP protein sequences from *Arabidopsis* and rice were used to identify the genes from the Chinese Spring IWGSC RefSeq v1.1 reference genome assembly (Ensembl Plants; https://plants.ensembl.org/Triticum_aestivum/Info/Index) with the local blast program (*E*-value <1e^− 10^). After removing duplicate searches using the CD-hit program, we identified the rest of the protein sequences with the Simple Modular Architecture Research Tool (SMART; http://smart.embl-heidelberg.de/smart/set_mode.cgi?NORMAL=1). We performed a phylogenetic analysis to filter the genes from TPP proteins previously identified from *Brachypodium distachyon*, *Populus trichocarpa* (poplar), *Arabidopsis thaliana*, *Oryza sativa* (rice), and *Zea mays* (maize). We also used phylogenetic analysis to sort the various TPP subfamilies and named the *TPP* genes from each of three wheat subgenomes (A, B, and D genomes) *TaTPPX_ZA*, *TaTPPX_ZB*, or *TaTPPX_ZD*, respectively, where X is the gene number and Z indicates its location on the wheat chromosome. The theoretical pI (isoelectric point) and Mw (molecular weight) of each putative wheat TPP protein were calculated using ExPasy (http://web.expasy.org/compute_pi/).

### Systematic analysis of the bioinformatics of the wheat TPP family

In order to perform a phylogenetic analysis of the wheat TPP family along with other species of plants, we obtained proteomes of *Populus trichocarpa* (poplar), *Brachypodium distachyon*, *Arabidopsis thaliana*, *Zea mays* (maize), and *sativa* (rice) from JGI (https://phytozome.jgi.doe. gov/pz/portal.html). We acquired all of the TPP protein sequences either directly from the supplemental materials or from the proteomes based on the gene locus, as indicated by other papers. We used the ClustalW program (default settings) to produce the multiple sequence alignments [46], and produced unrooted phylogenetic trees using the neighbor-joining (NJ) method and the MEGA6.0 software, using the full-length of the TPP protein sequences [47]. We used 1000 replications to estimate the bootstrap probability of each branch. We acquired information on the gene structure of *TaTPP* genes from the Chinese Spring IWGSC RefSeq v1.1 reference genome, which were analyzed with the Gene Structure Display Server 2.0 (GSDS; http://gsds.cbi.pku.edu.cn/). We also identified the conserved motifs of TaTPPs with the MEME program (http://meme-suite.org/), while we used the circlize package in R to assess the chromosomal distribution and draw the collinearity map [48]. We analyzed the *cis*-acting regulatory elements using plantCARE (http://bioinformatics.psb.ugent.be/webtools/plantcare/html/) and PLACE (https://www.dna.affrc.go.jp/PLACE/?action=newplace).

### Using RNA-seq data to analyze gene expression

We obtained RNA-seq data from ten different tissues, including the leaves, roots, and stems of five-leaf stage wheat seedlings, spikes at the heading stage, young spikes at early booting stage, flag leaves at the heading stage, and the grains of 5, 10, 15 and 20 DPA in order to analyze how *TaTPP* genes were expressed in different tissues (http://genedenovoweb.ticp.net:81/Wheat_GDR1246/index.php?m=index&f=index). We used Cufflinks and TopHat to assess gene expression, based on the RNA-seq data [49, 50] and calculated the FPKM value (fragments per kilobase of transcript per million fragments mapped) for each *TaTPP* gene. To produce the heat map, we used the log_10_-transformed (FPKM + 1) values of the *TaTPP* genes.

### Subcellular localization

We generated green fluorescent protein (GFP) expression vectors (CaMV35S*-GFP-NOS*) to analyze the subcellular localization of the TaTPP proteins. PCR and gene-specific primers were used to amplify the coding regions of *TaTPP6*, *TaTPP7*, *TaTPP9*, and *TaTPP11*, which were independently connected to the N-terminus of GFP in the expression vector. We isolated the wheat protoplasts from the mesophyll tissue of 2-week-old wheat seedlings, and used the PEG transfection method, along with the plasmid DNA of 35S*::TaTPP6-GFP*, 35S*::TaTPP7-GFP*, 35S*::TaTPP9-GFP*, 35S*::TaTPP11-GFP*, and 35S*::GFP* control, as described previously, for transformation [51]. Following PEG transfection, we incubated the wheat protoplasts in W5 solution (2 mM MES, 154 mM NaCl, 125 mM CaCl_2_, and 5 mM KCl, pH = 5.7) in a dark chamber for 18 h at 23 °C, and observed GFP fluorescence using a laser-scanning confocal microscope (FV3000, Olympus, Japan).

### *Arabidopsis* transformation and *TaTPP11* isolation

We obtained the *Arabidopsis* ecotype Columbia from Professor Zhensheng Kang’s Lab (Northwest A&F University, China), which we used to transform *TaTPP11*. The full-length opening reading frame of *TaTPP11* was amplified from the wheat variety Chinese Spring with gene-specific primers that were closed using the cauliflower mosaic virus (CaMV) 35S promoter, into the pGreen0029-GFP vector. The recombinant vector (35S*::TaTPP11-7D*) was then introduced into *Agrobacterium tumefaciens* GV3101 strain, after which the floral dip method [52] was used to turn it into *Arabidopsis* (*Arabidopsis thaliana*; ecotype Columbia). We placed the T_1_ seeds in an MS medium with 2% sucrose and 50 mg/mL kanamycin, allowing us to identify the transformants. Homozygous T_3_ plants were used to analyze the phenotype.

### Phylogenetic analysis and multiple sequence alignments

A phylogenetic analysis was performed on the full-length protein sequences from the TPP proteins of different plant species. We used MEGA (v6.0) software and the Neighbor-Joining (NJ) algorithm with 1000 bootstrap re-samplings to construct the phylogenetic tree, while the ClustalW software was used to conduct multiple sequence alignments, which were manually edited with BioEdit (v7.1).

### Statistical analyses

Each experiment was performed in triplicate, and data is presented and was analyzed after calculating the mean ± standard deviation (SD) of each experiment. A Student’s *t*-test was used to assess the statistical differences, while *P* < 0.05 was considered statistically significant and *P* < 0.01 was considered extremely statistically significant.

## Supplementary Information

**Additional file 1: Table S1.** All protein sequences used by this study.

**Additional file 2: Figure S1.** Phylogenetic tree of TPP proteins from *Populus*, *Arabidopsis*, rice, wheat, maize, and *B. distachyum*.

**Additional file 3: Figure S2.** Sequence logos for 20 motifs.

**Additional file 4: Figure S3.** Multiple sequence alignment of 31 TaTPPs. Identical amino acids are shaded black, while similar amino acids are shaded gray.

**Additional file 5: Table S2.** Organization of *cis*-acting regulatory elements in wheat *TaTPP* gene family promoters.

**Additional file 6: Figure S4.** Protein sequence alignment for three TaTPP11 homeologs.

**Additional file 7: Figure S5.** RT-PCR identification of *Arabidopsis* lines overexpressing *TaTPP11*.

**Additional file 8: Table S3.** Primers used in this research.

## Data Availability

All data generated or analyzed during this study are included in this article and its supplementary information files. However, the sequence data in this study can also be accessed at http://genedenovoweb.ticp.net:81/Wheat_GDR1246/index.php?m=index&f=index. In addition, all databases used in this study are open for public and the links are as follows: Chinese Crop Germplasm Resources Information System: http://www.cgris.net/zhongzhidinggou/index.php Ensembl Plants: https://plants.ensembl.org/Triticum_aestivum/Info/Index ExPasy: http://web.expasy.org/compute_ pi/. GSDS: http://gsds.cbi.pku.edu.cn/ JGI: https://phytozome.jgi.doe.gov/pz/portal.html MEME: http://meme-suite.org/ plantCARE: http://bioinformatics.psb.ugent.be/webtools/plantcare/html/ SMART: http://smart.embl-heidelberg.de/smart/set_mode.cgi?NORMAL=1

## Abbreviations

ABA: Abscisic acid
BLAST: Basic local alignment search tool
CDS: Coding sequences
DRE: Dehydration-responsive element
GSDS: Gene Structure Display Server
LTR: Low temperature responsive element
MBS: MYB binding site involved in drought-inducibility
MEME: Multiple Expectation Maximization for Motif Elicitation
MYB: MYB recognition site
MYC: MYC recognition site
pI: Isoelectric point
SMART: Simple Modular Architecture Research Tool

## References

[1] Paul MJ, Gonzalez-Uriarte A, Griffiths CA, Hassani-Pak K (2018). The role of trehalose 6-phosphate in crop yield and resilience. Plant Physiol.

[2] Fichtner F, Lunn JE (2021). The role of trehalose 6-phosphate (Tre6P) in plant metabolism and development. Annu Rev Plant Biol.

[3] Garg AK, Kim JK, Owens TG, Ranwala AP, Choi YD, Kochian LV (2002). Trehalose accumulation in rice plants confers high tolerance levels to different abiotic stresses. Proc Natl Acad Sci U S A.

[4] Paul MJ, Primavesi LF, Jhurreea D, Zhang Y (2008). Trehalose metabolism and signaling. Annu Rev Plant Biol.

[5] Mu M, Lu XK, Wang JJ, Wang DL, Yin ZJ, Wang S (2016). Genome-wide identification and analysis of the stress-resistance function of the *TPS* (Trehalose-6-phosphate synthase) gene family in cotton. BMC Genet.

[6] Yadav UP, Ivakov A, Feil R, Duan GY, Walther D, Giavalisco P (2014). The sucrose-trehalose 6-phosphate (Tre6P) nexus: specificity and mechanisms of sucrose signalling by Tre6P. J Exp Bot.

[7] Wingler A (2002). The function of trehalose biosynthesis in plants. Phytochemistry..

[8] Zeid IM (2009). Trehalose as osmoprotectant for maize under salinity-induced stress. Res J Agric Biol Sci.

[9] Ali Q, Ashraf M (2011). Induction of drought tolerance in maize (*Zea mays* L.) due to exogenous application of trehalose: growth, photosynthesis, water relations and oxidative defence mechanism. J Agron Crop Sci.

[10] Julieta RS, Ramón S, Jesús C, Gabriel I (2010). Trehalose accumulation in *Azospirillum brasilense* improves drought tolerance and biomass in maize plants. Fems Microbiol Lett.

[11] Macovei A, Pagano A, Cappuccio M, Gallotti L, Dondi D (2019). A snapshot of the trehalose pathway during seed imbibition in *medicago truncatula* reveals temporal- and stress-dependent shifts in gene expression patterns associated with metabolite changes. Front Plant Sci.

[12] Satoh-Nagasawa N, Nagasawa N, Malcomber S, Sakai H, Jackson D (2006). A trehalose metabolic enzyme controls inflorescence architecture in maize. Nature..

[13] Carillo P, Feil R, Gibon Y, Satoh-Nagasawa N (2013). A fluorometric assay for trehalose in the picomole range. Plant Methods.

[14] Nuccio ML, Wu J, Mowers R, Zhou HP, Meghji M, Primavesi LF (2015). Expression of trehalose-6-phosphate phosphatase in maize ears improves yield in well-watered and drought conditions. Nat Biotechnol.

[15] Figueroa CM, Lunn JE (2016). A tale of two sugars: trehalose 6-phosphate and sucrose. Plant Physiol.

[16] Griffiths CA, Sagar R, Geng Y, Primavesi LF, Patel MK, Passarelli MK (2016). Chemical intervention in plant sugar signalling increases yield and resilience. Nature..

[17] Wahl V, Ponnu J, Schlereth A, Arrivault S, Langenecker T, Franke A (2013). Regulation of flowering by trehalose-6-phosphate signaling in *Arabidopsis thaliana*. Science..

[18] Hulsmans S, Rodriguez M, Coninck BD, Rolland F (2016). The *SnRK1* energy sensor in plant biotic interactions. Trends Plant Sci.

[19] Zhang Y, Primavesi LF, Jhurreea D, Andralojc PJ, Mitchell RA, Powers SJ (2009). Inhibition of SNF1-related protein kinase1 activity and regulation of metabolic pathways by trehalose-6-phosphate. Plant Physiol.

[20] Martínez-Barajas E, Delatte T, Schluepmann H, de Jong GJ, Somsen GW, Nunes C (2011). Wheat grain development is characterized by remarkable trehalose 6-phosphate accumulation pregrain filling: tissue distribution and relationship to SNF1-related protein kinase1 activity. Plant Physiol.

[21] Cabib E, Leloir LF (1958). The biosynthesis of trehalose phosphate. J Biol Chem.

[22] Avonce N, Mendoza-Vargas A, Morett E, Iturriaga G (2006). Insights on the evolution of trehalose biosynthesis. BMC Evol Biol.

[23] Lunn JE (2007). Gene families and evolution of trehalose metabolism in plants. Funct Plant Biol.

[24] Dijck PV (2010). The Cytophaga hutchinsonii ChTPSP: first characterized bifunctional TPS-TPP protein as putative ancestor of all eukaryotic trehalose biosynthesis proteins. Mol Biol Evol.

[25] Yang HL, Liu YJ, Wang CL, Zeng QY, Natarajan K (2012). Molecular evolution of trehalose-6-phosphate synthase (*TPS*) gene family in populus, *Arabidopsis* and rice. PLoS One.

[26] Vandesteene L, Lopez-Galvis L, Vanneste K, Feil R, Maere S, Lammens W (2012). Expansive evolution of the trehalose-6-phosphate phosphatase gene family in *Arabidopsis*. Plant Physiol.

[27] Krasensky J, Broyart C, Rabanal FA, Jonak C (2014). The redox-sensitive chloroplast trehalose-6-phosphate phosphatase *AtTPPD* pegulates salt stress tolerance. Antioxid Redox Signal.

[28] Lin Q, Yang J, Wang Q, Zhu H, Wang K (2019). Overexpression of the trehalose-6-phosphate phosphatase family gene *AtTPPF* improves the drought tolerance of *Arabidopsis thaliana*. BMC Plant Biol.

[29] Lin Q, Wang S, Yihang D, Wang J, Wang K (2020). *Arabidopsis thaliana* trehalose-6-phosphate phosphatase gene *TPPI* enhances drought tolerance by regulating stomatal apertures. J Exp Bot.

[30] Pramanik M, Imai R (2005). Functional identification of a trehalose 6-phosphate phosphatase gene that is involved in transient induction of trehalose biosynthesis during chilling stress in rice. Plant Mol Biol.

[31] Shima M, Tahara I (2007). Biochemical characterization of rice trehalose-6-phosphate phosphatases supports distinctive functions of these plant enzymes. FEBS J.

[32] Kretzschmar T, Pelayo M, Trijatmiko KR, Gabun AAL, Alam R, Jimenez R (2015). A trehalose-6-phosphate phosphatase enhances anaerobic germination tolerance in rice. Nat Plants..

[33] Claeys H, Vi SL, Xu X, Satoh-Nagasawa N, Eveland AL, Goldshmidt A (2019). Control of meristem determinacy by trehalose 6-phosphate phosphatases is uncoupled from enzymatic activity. Nat Plants.

[34] Appels R, Eversole K, Feuillet C, Keller B, Rogers J, Stein N (2018). Shifting the limits in wheat research and breeding using a fully annotated reference genome. Science..

[35] Holub EB (2001). The arms race is ancient history in *Arabidopsis*, the wildflower. Nat Rev Genet.

[36] Xu G, Guo C, Shan H, Kong H (2012). Divergence of duplicate genes in exon-intron structure. Pro Nat Acad Sci USA.

[37] Lescot M (2002). PlantCARE, a database of plant cis-acting regulatory elements and a portal to tools for in silico analysis of promoter sequences. Nucleic Acids Res.

[38] Yamaguchi-Shinozaki K, Shinozaki K (2005). Organization of *cis*-acting regulatory elements in osmotic- and cold-stress-responsive promoters. Trends Plant Sci.

[39] Feldman M, Levy AA (2005). Allopolyploidy - a shaping force in the evolution of wheat genomes. Cytogenet Genome Res.

[40] Marcussen T, Sandve SR, Heier L, Spannagl M, Pfeifer M, Jakobsen KS (2014). Ancient hybridizations among the ancestral genomes of bread wheat. Science..

[41] Flagel LE, Wendel JF (2009). Gene duplication and evolutionary novelty in plants. New Phytol.

[42] Li Z, Zhang C, Guo Y, Niu W, Wang Y, Xu Y. Evolution and expression analysis reveal thepotential role of the *HD-zip* gene family in regulation of embryo abortion in grapes (*Vitis vinifera* L.). BMC Genomics. 2017;18(1):–744. 10.1186/s12864-017-4110-y PMC5609062 28934927

[43] Zhao ML, Ni J, Chen MS, Xu ZF (2019). Ectopic expression of *Jatropha curcas TREHALOSE-6-PHOSPHATE PHOSPHATASE J* causes late-flowering and heterostylous phenotypes in *Arabidopsis* but not in *Jatropha*. Int J Mol Sci.

[44] Ruan J, Zhou Y, Zhou M, Yan J, Khurshid M, Weng W (2019). Jasmonic acid signaling pathway in plants. Int J Mol Sci.

[45] Livak KJ, Schmittgen TD (2001). Analysis of relative gene expression data using real-time quantitative PCR and the 2(−Delta Delta C(T)) method. Methods..

[46] Thompson J, Gibson T, Higgins D. Multiple sequence alignment using ClustalW and ClustalX. Curr Protoc Bioinformatics. 2002; chapter2, Unit 2.3. 10.1002/0471250953.bi0203s00 18792934

[47] Tamura K, Stecher G, Peterson D, Filipski A, Kumar S (2013). MEGA6: molecular evolutionary genetics analysis version 6.0. Mol Biol Evol.

[48] Gu Z, Gu L, Eils R, Schlesner M, Brors B (2014). Circlize implements and enhances circular visualization in R. Bioinformatics..

[49] Trapnell CRA, Goff L, Pertea G, Kim D, Kelley DR (2012). Differential gene and transcript expression analysis of RNA-seq experiments with TopHat and cufflinks. Nat Protoc.

[50] Trapnell C, Hendrickson DG, Sauvageau M, Goff L, Rinn JL, Pachter L (2013). Differential analysis of gene regulation at transcript resolution with RNA-seq. Nat Biotechnol.

[51] Yoo SD, Cho YH, Sheen J (2007). *Arabidopsis* mesophyll protoplasts: a versatile cell system for transient gene expression analysis. Nat Protoc.

[52] Clough SJ, Bent AF (1998). Floral dip: a simplified method for *Agrobacterium*-mediated transformation of *Arabidopsis thaliana*. Plant J.

