# Supplementary figures and images for: Genome-wide analysis of trehalose-6-phosphate phosphatases (TPP) gene family in wheat indicates their roles in plant development and stress response
Source: BMC Plant Biol. 2022 Mar 16;22:120. doi: 10.1186/s12870-022-03504-0 (PMC8925099; doi:10.1186/s12870-022-03504-0)

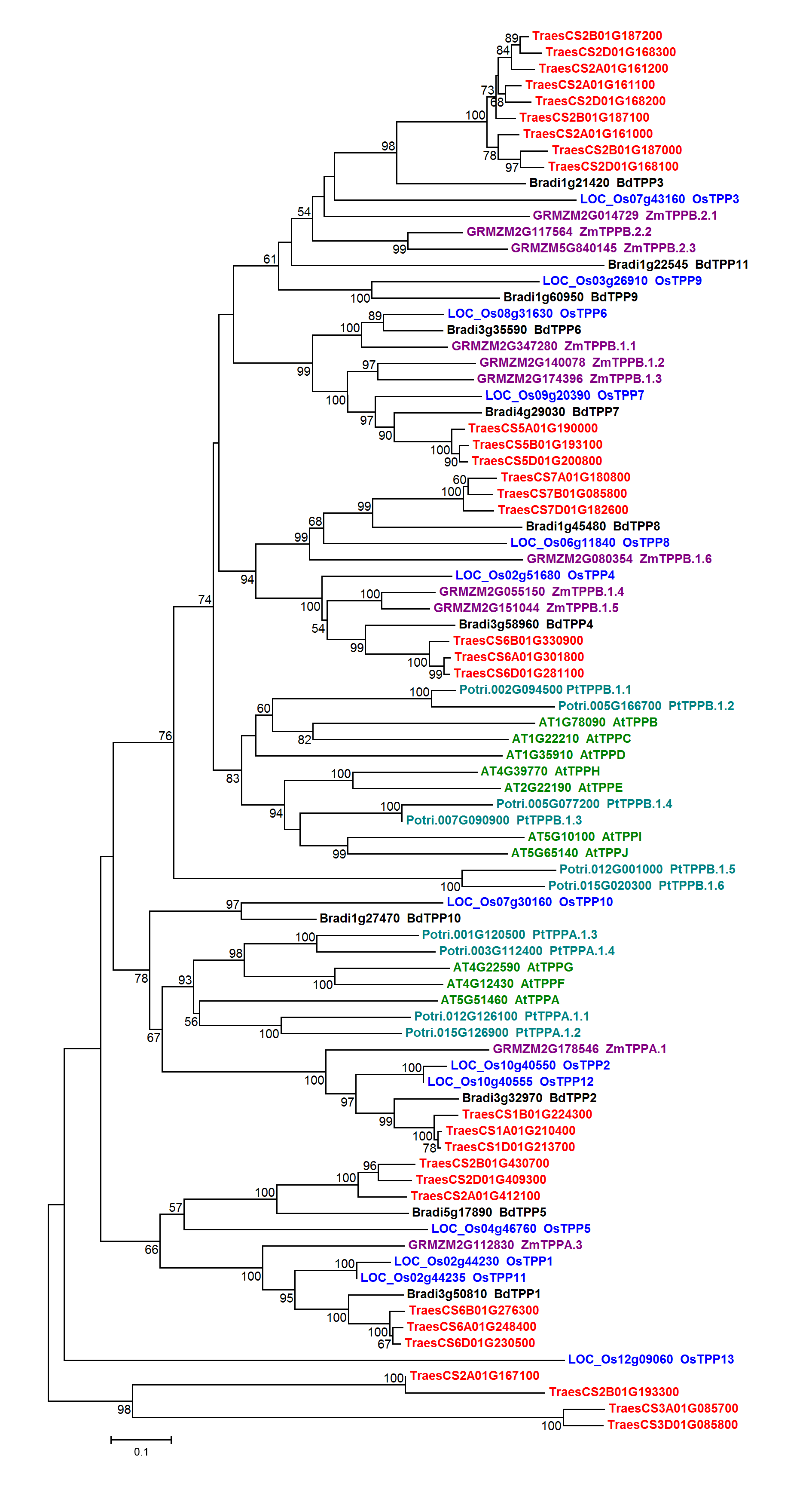

Supplement: Supplementary file 2 — Additional file 2: Figure S1. Phylogenetic tree of TPP proteins from Populus, Arabidopsis, rice, wheat, maize, and B. distachyum. [file 12870_2022_3504_MOESM2_ESM.tif]

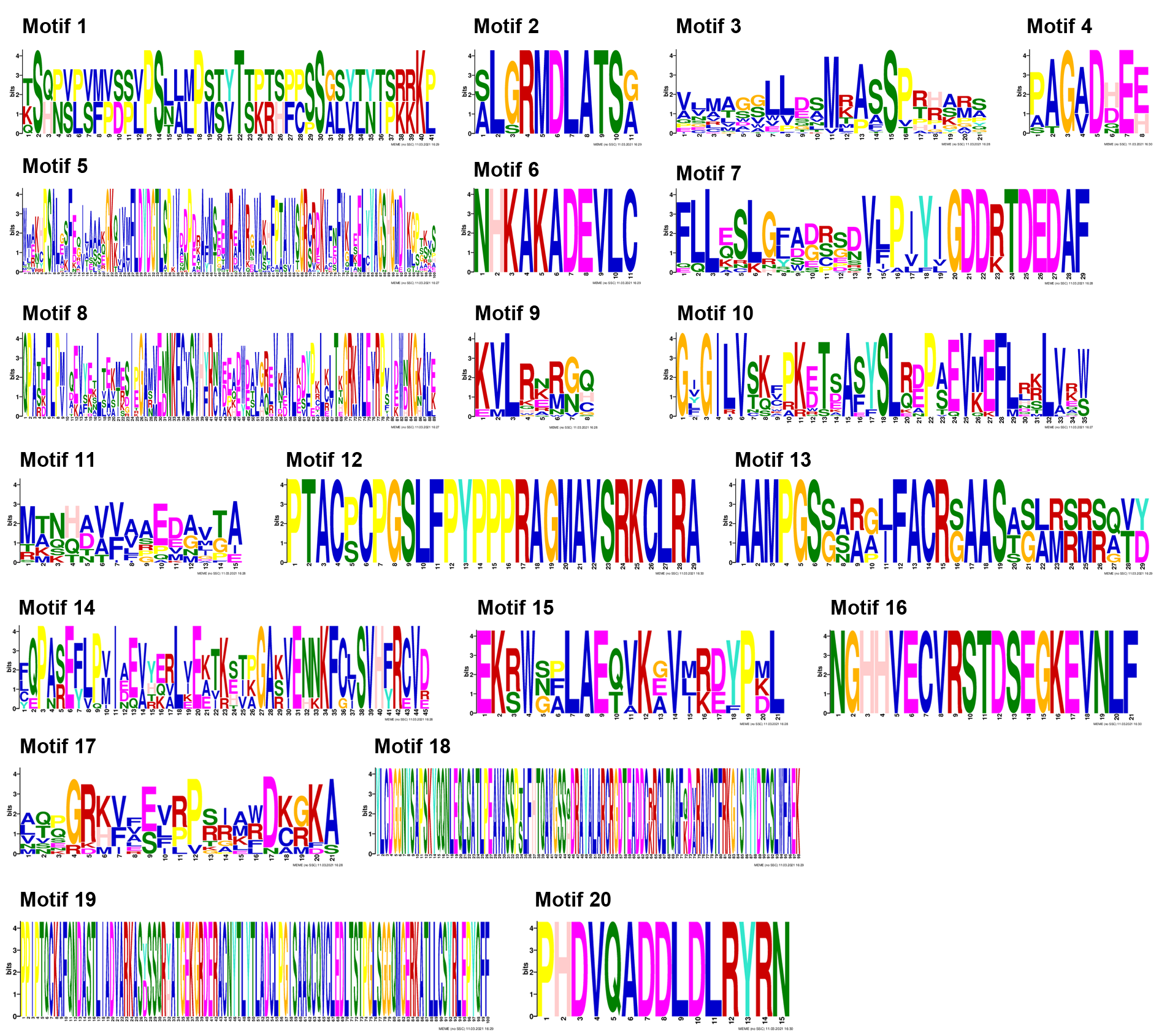

Supplement: Supplementary file 3 — Additional file 3: Figure S2. Sequence logos for 20 motifs. [file 12870_2022_3504_MOESM3_ESM.tif]

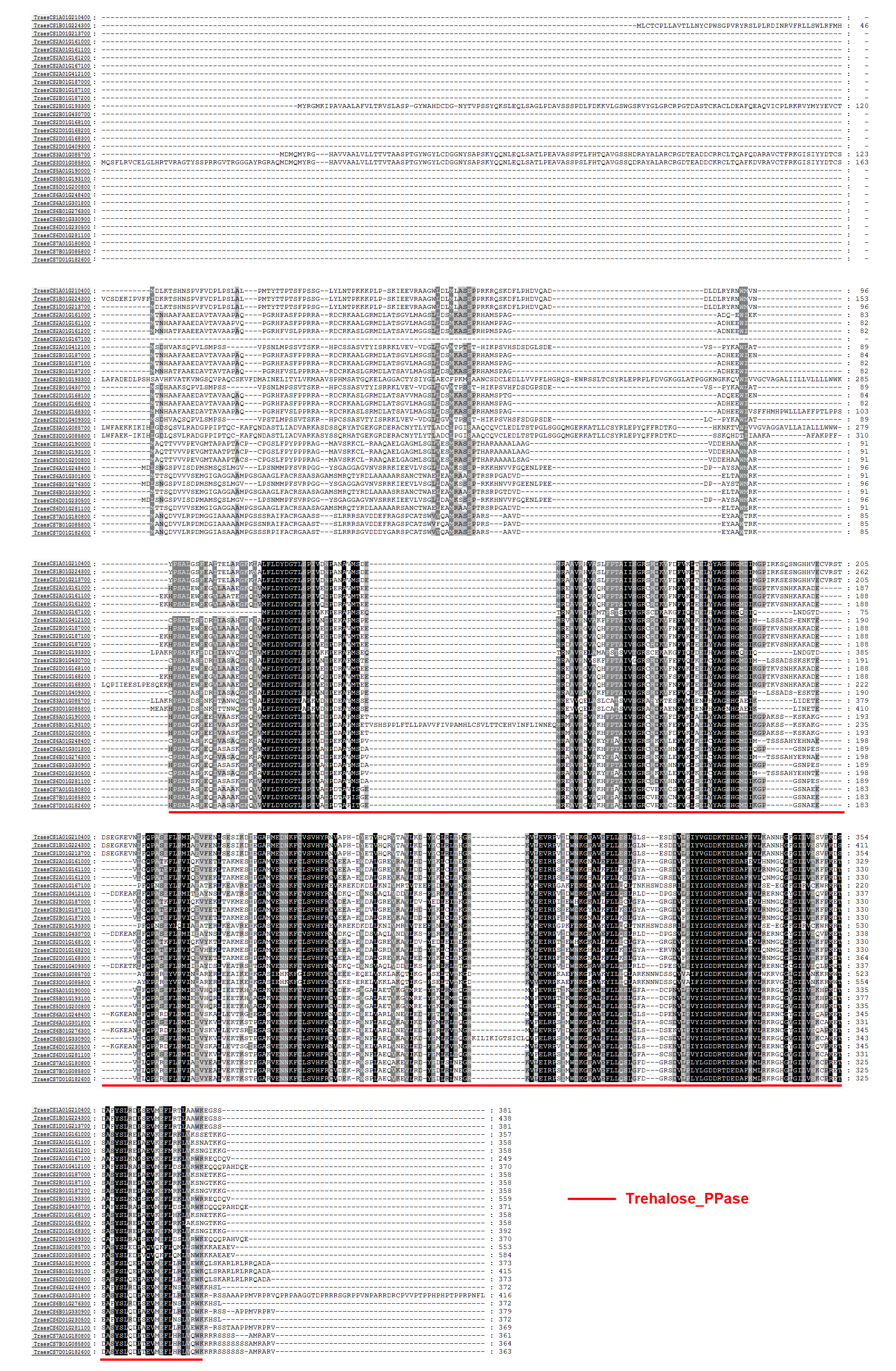

Supplement: Supplementary file 4 — Additional file 4: Figure S3. Multiple sequence alignment of 31 TaTPPs. Identical amino acids are shaded black, while similar amino acids are shaded gray. [file 12870_2022_3504_MOESM4_ESM.tif]

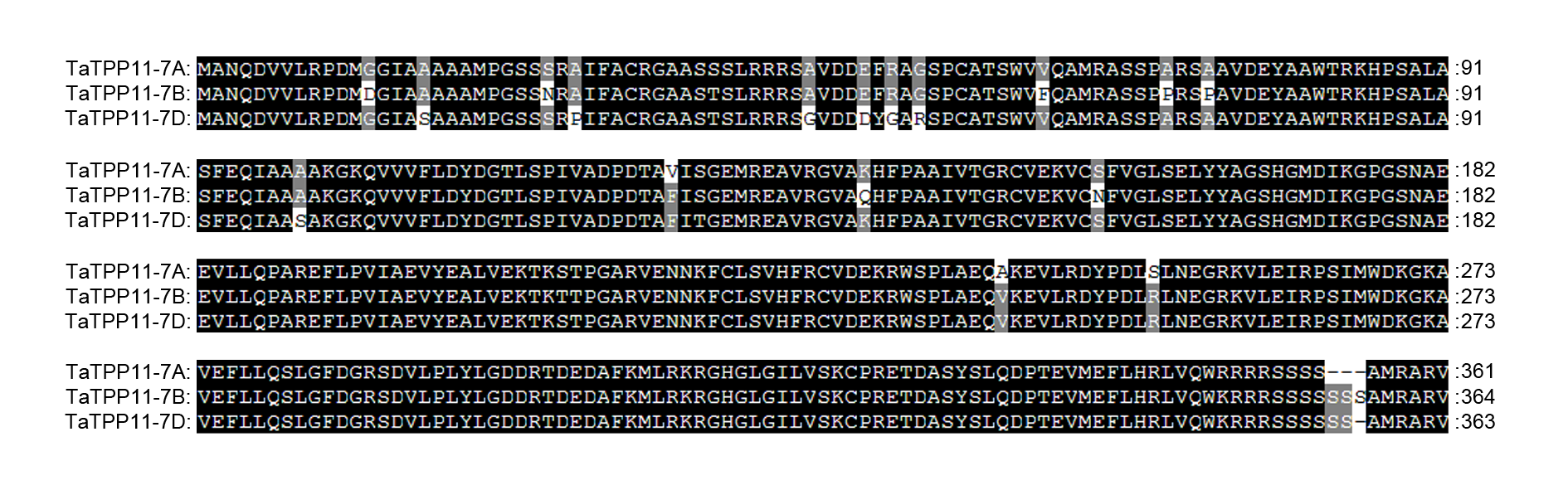

Supplement: Supplementary file 6 — Additional file 6: Figure S4. Protein sequence alignment for three TaTPP11 homeologs. [file 12870_2022_3504_MOESM6_ESM.tif]

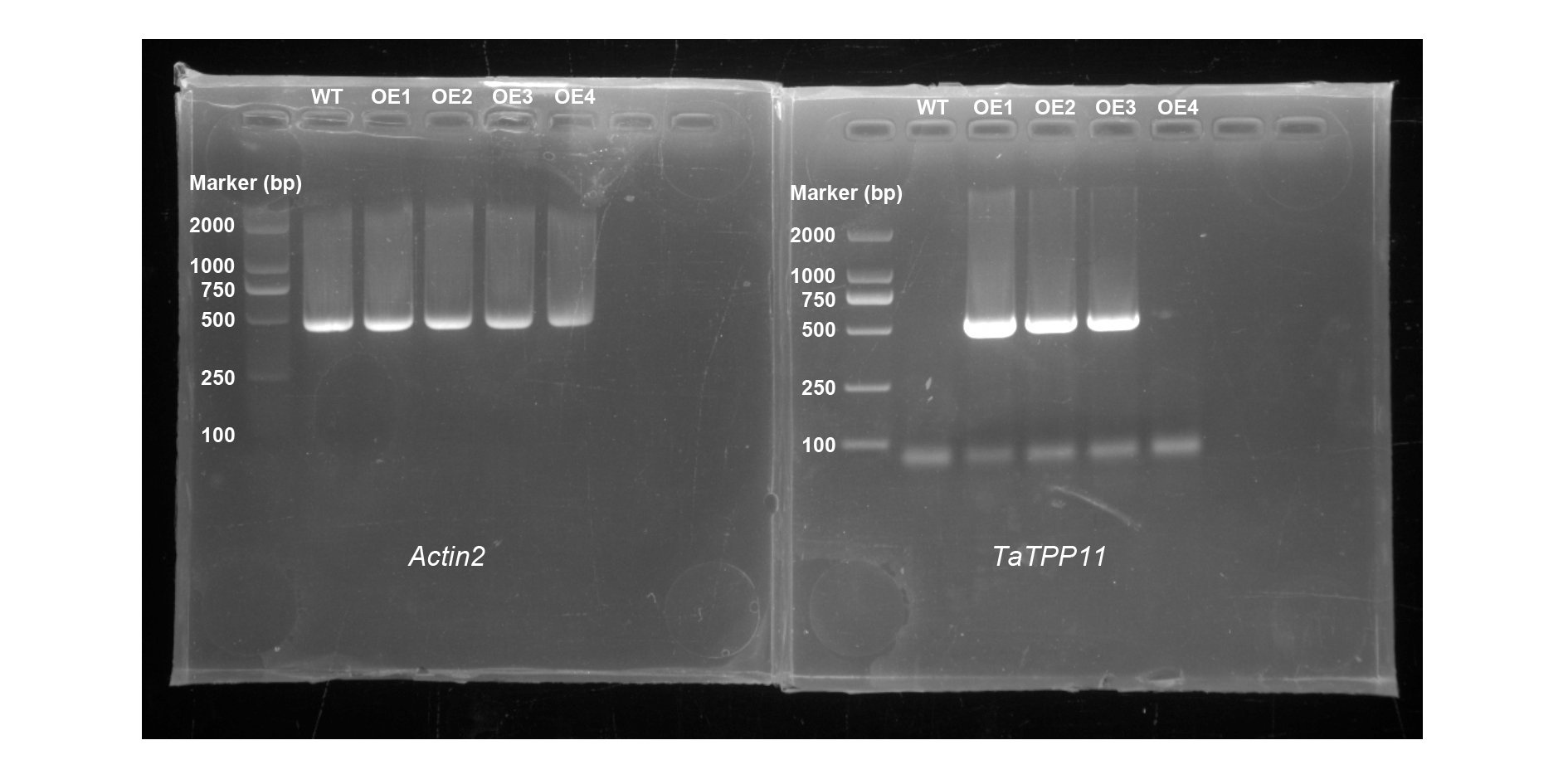

Supplement: Supplementary file 7 — Additional file 7: Figure S5. RT-PCR identification of Arabidopsis lines overexpressing TaTPP11. [file 12870_2022_3504_MOESM7_ESM.tif]
